# Independence estimators for re-randomisation trials in multi-episode settings: a simulation study

**DOI:** 10.1186/s12874-021-01433-4

**Published:** 2021-10-30

**Authors:** Brennan C. Kahan, Ian R. White, Sandra Eldridge, Richard Hooper

**Affiliations:** 1 MRC Clinical Trials Unit at UCL, London, UK; 2 Pragmatic Clinical Trials Unit, Queen Mary University of London, London, UK

**Keywords:** Re-randomisation, Re-randomisation trials, Independence estimators, Simulation study, Estimands

## Abstract

**Background:**

Re-randomisation trials involve re-enrolling and re-randomising patients for each new treatment episode they experience. They are often used when interest lies in the average effect of an intervention across all the episodes for which it would be used in practice. Re-randomisation trials are often analysed using independence estimators, where a working independence correlation structure is used. However, research into independence estimators in the context of re-randomisation has been limited.

**Methods:**

We performed a simulation study to evaluate the use of independence estimators in re-randomisation trials. We focussed on a continuous outcome, and the setting where treatment allocation does not affect occurrence of subsequent episodes. We evaluated different treatment effect mechanisms (e.g. by allowing the treatment effect to vary across episodes, or to become less effective on re-use, etc), and different non-enrolment mechanisms (e.g. where patients who experience a poor outcome are less likely to re-enrol for their second episode). We evaluated four different independence estimators, each corresponding to a different estimand (per-episode and per-patient approaches, and added-benefit and policy-benefit approaches).

**Results:**

We found that independence estimators were unbiased for the per-episode added-benefit estimand in all scenarios we considered. We found independence estimators targeting other estimands (per-patient or policy-benefit) were unbiased, except when there was differential non-enrolment between treatment groups (i.e. when different types of patients from each treatment group decide to re-enrol for subsequent episodes). We found the use of robust standard errors provided close to nominal coverage in all settings where the estimator was unbiased.

**Conclusions:**

Careful choice of estimand can ensure re-randomisation trials are addressing clinically relevant questions. Independence estimators are a useful approach, and should be considered as the default estimator until the statistical properties of alternative estimators are thoroughly evaluated.

**Supplementary Information:**

The online version contains supplementary material available at 10.1186/s12874-021-01433-4.

## Introduction

Re-randomisation trials can be used to evaluate interventions in multi-episode settings, where some patients require treatment on more than one occasion [1–6]. In re-randomisation trials, patients are re-enrolled and re-randomised for each new treatment episode they experience (providing they continue to remain eligible). The number of times each patient is enrolled is not specified by the design, but instead is based on the number of treatment episodes they experience during the trial [4]. The two key design requirements for re-randomisation trials are that (i) patients are only re-enrolled when the follow-up period from their previous enrolment is complete; and (ii) randomisations for the same patient are independent [4].

The use of re-randomisation can increase efficiency, as a larger number of available treatment episodes are enrolled [3–5], and can also help to address questions about the average effect of the intervention across all episodes for which it would be used in practice [1]. Re-randomisation trials have been used to evaluate interventions for sickle cell pain crises (where patients are re-randomised for each new pain crisis) [7], severe asthma exacerbations (where patients are re-randomised for each new exacerbation) [8], influenza vaccines (where patients are re-randomised for each new influenza seasons) [9], in-vitro fertilisation (where participants are re-randomised for each new cycle) [10], and pre-term birth (where participants are re-randomised for each new pregnancy) [11].

Independence estimators have been proposed for re-randomisation trials, where a working independence correlation structure is used [1, 4], and this approach is commonly used in practice [5]. However, prior methodological work around these estimators is limited. First, previous work has primarily focussed on the setting where the treatment effect is the same across all patients and episodes [2, 4, 6]. However, this may not always be the case in practice; for instance, the intervention may become more or less effective the more often it is used, or patients with more severe underlying conditions may be both predisposed to experience a larger number of episodes, and always have worse responses to treatment.

Second, most research has been in the setting where there is no differential non-enrolment (i.e. in the setting where the same type of patients from each treatment group re-enrols for subsequent episodes). However, this may not always be the case in practice; for instance, in open-label trials where patients are aware of their treatment allocation, their probability of re-enrolling may be affected by the combination of prior treatment allocation as well as their response to treatment.

Third, the use of robust standard errors which allow for clustering have been proposed [1], however their use has never been empirically evaluated for re-randomisation trials.

Finally, most previous literature has focussed on an estimator which targets a per-episode added-benefit estimand, which represents the average treatment effect across episodes, over and above any benefit from previous assignment to the intervention [1]. This evaluation is warranted, as this is the estimator which is most commonly used in practice [5]; however, other estimands have been proposed [1], and it would be of interest to evaluate the use of independence estimators for these alternate estimands.

The purpose of this paper is to comprehensively evaluate the use of independence estimators in re-randomisation trials using a large simulation study. In particular, we will evaluate their use (i) for different estimands; (ii) under non-constant treatment effect mechanisms; (iii) under differential non-enrolment; and (iv) in conjunction with robust standard errors. For simplicity, we focus on a setting where patients experience a maximum of two episodes, and where the outcome of interest is continuous. We also focus on the setting where the interventions under study do not affect whether future episodes occur (i.e. patients would experience the same number of episodes during the trial period regardless of which treatments they are allocated to), though we note re-randomisation trials can also be used when this is not the case.

## Methods

### Notation

We briefly summarise some of the notation that will be used in this article. Some further notation is introduced in later sections as required.

Let *i* index patient, and *j* index the episode number within the trial. Then, let *Y*_*ij*_ denote the outcome for patient *i* at episode *j*, and *Z*_*ij*_ denote their treatment allocation (0 = control, 1 = intervention). ${\overset{\sim }{Z}}_{ij}$ is the patient’s ‘treatment history’, which denoates their treatment allocations in their previous episodes (e.g. ${\overset{\sim }{Z}}_{13}$ would be the vector (*Z*_11_, *Z*_12_)). Then, let $Y_{ij}^{\left(Z_{ij}=0,\widetilde{Z_{ij}}=\widetilde{Z_{ij}}\right)}$ represent the potential outcome under *Z* = 0 and treatment history $\overset{\sim }{Z_{ij}}=\overset{\sim }{z_{ij}}$. For clarity, we drop subscripts inside the brackets, as these are the same as subscripts on the outside of the brackets; for example, $Y_{ij}^{\left(Z=1,\widetilde Z=\widetilde z\right)}$ is the same as $Y_{ij}^{\left(Z_{ij}=1,\widetilde{Z_{ij}}=\widetilde{Z_{ij}}\right)}$ .

Finally, let *M*_*i*_ be the number of episodes for which patient *i* is enrolled in the trial, *M*_*T*_ be the total number of episodes enrolled, and *M*_*T*(*j*)_ be the total number of patients for whom *M*_*i*_ = *j* (i.e. the number of patients enrolled for *j* episodes). There are *N*_*T*_ total patients enrolled in the trial, and *N*_*j*_ represents the number of patients who are enrolled for at least *j* episodes (i.e. for whom M_i_ ≥ j).

### Simulation study

We conducted a large simulation study to evaluate the bias and coverage of independence estimators for re-randomisation trials [12]. This simulation study focussed on a setting where patients were enrolled in a trial for a maximum of two episodes. For most scenarios, we chose parameter values that are larger than those we might expect to see in practice; this was to ensure that if estimators were biased in any scenarios, we would be able to identify it.

We performed three different sets of simulations (labelled simulation study 1, 2a, and 2b). We describe the estimands, methods of analysis, and performance measures used across all three simulation studies below, and then describe the data generating models for each of the three simulation studies.

All simulations were conducted using Stata v15.1.

### Estimands

For all simulation scenarios, we used the following four estimands, which have been described previously [1]: (a) per-episode added-benefit; (b) per-patient added-benefit; (c) per-episode policy-benefit; and (d) per-patient policy-benefit.

Descriptions of these estimands are available in Table 1, and are described briefly in the sections below; full details are available in reference [1]. We calculated the true value of each estimand for each simulation scenario using the method described in reference [1]; for simulation scenario 1, we calculated these values analytically, and for simulation scenarios 2a and 2b, we calculated these by simulating a single large dataset (this process is described further below); the reason we used simulation for scenarios 2a and 2b was because they involved non-enrolment (i.e. some patients did not re-enrol for their 2nd episode), which made the analytical calculations more challenging.

**Table 1:** Summary of estimands and estimators. Policy-benefit estimators are based on setting with maximum of two episodes per patient

Estimand	Definition	Description	Estimator
Per-episode added-benefit	${\upbeta}_E^{AB}=E\left({Y}_{(IJ)^E}^{\left(Z=1,\overset{\sim }{Z}\right)}-{Y}_{(IJ)^E}^{\left(Z=0,\overset{\sim }{Z}\right)}\right)$	Provides the additional effect of being assigned the intervention in the current episode, over and above the benefit of being assigned the intervention in previous episodes Provides an average effect across episodes	${\hat{\upbeta}}_E^{AB}=\frac{\sum_{ij}{Y}_{ij}{Z}_{ij}}{\sum_{ij}{Z}_{ij}}-\frac{\sum_{ij}{Y}_{ij}\left(1-{Z}_{ij}\right)}{\sum_{ij}\left(1-{Z}_{ij}\right)}$
Per-episode policy-benefit	${\upbeta}_E^{PB}=E\left({Y}_{(IJ)^E}^{\left(Z=1,\overset{\sim }{Z}=\overset{\sim }{1}\right)}-{Y}_{(IJ)^E}^{\left(Z=0,\overset{\sim }{Z}=\overset{\sim }{0}\right)}\right)$	Provides the effect of a treatment policy where patients are assigned intervention vs. control for all episodes Provides an average effect across episodes	Step 1: ${Y}_{ij}=\upalpha +\upbeta {Z}_{ij}+\upgamma {Z}_{i,j-1}+\updelta {Z}_{ij}{Z}_{i,j-1}+{\beta}_{ep}{X}_{e{p}_{ij}}+{\upvarepsilon}_{ij}$ Step 2: ${\hat{\upbeta}}_E^{PB}=\frac{N_1}{M_T}\left(\hat{\upbeta}\right)+\frac{N_2}{M_T}\left(\hat{\upgamma}+\hat{\upbeta}+\hat{\updelta}\right)$
Per-patient added-benefit	${\upbeta}_P^{AB}=E\left({Y}_{(IJ)^P}^{\left(Z=1,\overset{\sim }{Z}\right)}-{Y}_{(IJ)^P}^{\left(Z=0,\overset{\sim }{Z}\right)}\right)$	Provides the additional effect of being assigned the intervention in the current episode, over and above the benefit of being assigned the intervention in previous episodes Provides an average effect across patients	${\hat{\upbeta}}_P^{AB}=\frac{\sum_{ij}{W}_i{Y}_{ij}{Z}_{ij}}{\sum_{ij}{W}_i{Z}_{ij}}-\frac{\sum_{ij}{W}_i{Y}_{ij}\left(1-{Z}_{ij}\right)}{\sum_{ij}{W}_i\left(1-{Z}_{ij}\right)}$
Per-patient policy-benefit	${\upbeta}_P^{PB}=E\left({Y}_{(IJ)^P}^{\left(Z=1,\overset{\sim }{Z}=\overset{\sim }{1}\right)}-{Y}_{(IJ)^P}^{\left(Z=0,\overset{\sim }{Z}=\overset{\sim }{0}\right)}\right)$	Provides the effect of a treatment policy where patients are assigned intervention vs. control for all episodes Provides an average effect across patients	Step 1: ${Y}_{ij}=\upalpha +\upbeta {Z}_{ij}+\upgamma {Z}_{i,j-1}+\updelta {Z}_{ij}{Z}_{i,j-1}+{\beta}_{ep}{X}_{e{p}_{ij}}+{\upvarepsilon}_{ij}$ using weighted least squares, with weights ${W}_i=\frac{1}{M_i}$. Step 2: ${\hat{\upbeta}}_P^{PB}=\frac{M_{T(1)}}{N_T}\left(\hat{\upbeta}\right)+\frac{M_{T(2)}}{N_T}\left(\frac{1}{2}\hat{\upbeta}+\left(\frac{1}{2}\right)\left(\hat{\upbeta}+\hat{\upgamma}+\hat{\updelta}\right)\right)$

#### Per-episode added-benefit estimand

The per-episode added-benefit estimand is defined as:

$${\upbeta}_E^{AB}=E\left({Y}_{(IJ)^E}^{\left(Z=1,\overset{\sim }{Z}\right)}-{Y}_{(IJ)^E}^{\left(Z=0,\overset{\sim }{Z}\right)}\right)$$

where (*IJ*)^*E*^ represents a randomly selected episode from the trial (each with equal probability), and so ${Y}_{(IJ)^E}$ represents the outcome for a randomly selected episode.

In this estimand, each episode is given equal weight. It addresses the questions: what is the average treatment effect across episodes, over and above any benefit of the intervention from previous episodes? Broadly, it measures the benefit of the intervention conditional on a shared treatment history (i.e. it measures the difference in potential outcomes under intervention vs. control, where both potential outcomes share a common treatment history), and then takes a weighted average of this effect across the different treatment histories. Importantly, the treatment effect here measures the benefit conferred from treatment in the current episode, over and above any benefit carried forward from being assigned intervention in previous episodes.

#### Per-patient added-benefit estimand

The per-patient added-benefit estimand is defined as:

$${\upbeta}_P^{AB}=E\left({Y}_{(IJ)^P}^{\left(Z=1,\overset{\sim }{Z}\right)}-{Y}_{(IJ)^P}^{\left(Z=0,\overset{\sim }{Z}\right)}\right)$$

where (*IJ*)^*P*^ represents a randomly selected episode from a randomly selected patient (i.e. first a patient is randomly selected from the trial, each with equal probability; and then an episode from within that patient is selected, each with equal probability). Then ${Y}_{(IJ)^P}$ represents the outcome for randomly selected episode from a randomly selected patient.

In this estimand, each patient is given equal weight. It addresses the question: what is the average treatment effect across patients, over and above any benefit of the intervention from previous episodes? Broadly, it measures an average of the patient-specific treatment effects. As above, the treatment effect here measures the benefit conferred from treatment in the current episode, over and above any benefit carried forward from being assigned intervention in previous episodes.

#### Per-episode policy-benefit estimand

The per-episode policy-benefit estimand is defined as:

$${\upbeta}_E^{PB}=E\left({Y}_{(IJ)^E}^{\left(Z=1,\overset{\sim }{Z}=\overset{\sim }{1}\right)}-{Y}_{(IJ)^E}^{\left(Z=0,\overset{\sim }{Z}=\overset{\sim }{0.}\right)}\right)$$

where $\overset{\sim }{Z}=\overset{\sim }{1}$ denotes the patient has been assigned to intervention in all previous episodes (and vice versa for $\overset{\sim }{Z}=\overset{\sim }{0}$).

In this estimand, each episode is given equal weight. It addresses the question: what is the average treatment effect across episodes, where patients are assigned intervention for all episodes vs. control for all episodes? Broadly, it measures the difference in potential outcomes under intervention for this and all previous episodes vs. control for this and all previous episodes.

#### Per-patient policy-benefit estimand

The per-patient policy-benefit estimand is defined as:

$${\upbeta}_P^{PB}=E\left({Y}_{(IJ)^P}^{\left(Z=1,\overset{\sim }{Z}=\overset{\sim }{1}\right)}-{Y}_{(IJ)^P}^{\left(Z=0,\overset{\sim }{Z}=\overset{\sim }{0}\right)}\right)$$

In this estimand, each patient is given equal weight. It addresses the question: what is the average treatment effect across patients, where patients are assigned intervention for all episodes vs. control for all episodes? Broadly, it measures the difference in potential outcomes under intervention for this and all previous episodes vs. control for this and all previous episodes.

### Methods of analysis

We implemented four independence estimators, each corresponding to one of the four the estimands listed above. Briefly, we used a working independence correlation structure in conjunction with cluster-robust standard errors, with patients acting as the cluster [13]. Although working correlation structures are typically used in conjunction with generalised estimating equations, here we use them with linear regression models which implicitly assume an independence correlation structure. The main benefit to using linear regression models here is that inference can be based on the t-distribution, which is not the case with generalised estimating equations.

These estimators are fully described in the sections below, and a summary is provided in Table 1. A full overview of these estimators is provided in reference (1), and details of how these estimators were implemented in Stata is shown in Table 2.

**Table 2:** Stata code to implement independence estimators. ‘y’ denotes patient outcome, ‘z’ denotes treatment allocation, ‘id’ is a unique identifier for patient, ‘m_i’ denotes the number of episodes for which the patient is enrolled in the trial, ‘z_prev’ denotes the patient’s treatment allocation in their previous episode (and is set to 0 if it is the patient’s first episode), ‘x_ep’ is an indicator for episode 2, ‘prop_1st_ep’ and ‘prop_2nd_ep’ represent the proportion of episodes in the trial which are 1st and 2nd episodes respectively, and ‘prop_has_1ep’ and ‘prop_has_2ep’ denote the proportion of patients enrolled in the trial for one and two episodes respectively. In order to run the above code in Stata, ‘prop_1st_ep’, ‘prop_2nd_ep’, ‘prop_has_1ep’, and ‘prop_has_2ep’ must be saved as Stata local macros

Estimator	Stata code
*Added-benefit*	
Per-episode	reg y z, vce (cluster id)
Per-patient	reg y z [pw = 1/m_i], vce (cluster id)
*Policy-benefit*	
Per-episode	reg y z##z_prev x_ep, vce (cluster id) lincom `prop_1st_ep’*_b[1.z] + /// `prop_2nd_ep’*(_b[1.z] + _b[1.z_prev] + _b[1.z#1.z_prev])
Per-patient	reg y z##z_prev x_ep [pw = 1/m_i], vce (cluster id) lincom `prop_has_1ep’*(_b[1.z]) + `prop_has_2ep’*((1/2)*(_b[1.z]) + /// (1/2)*(_b[1.z] + _b[1.z_prev] + _b[1.z#1.z_prev]))

#### Per-episode added-benefit estimator

We used the following estimator, which corresponds to a simple difference in means between all intervention episodes vs. all control episodes:

1 $${\hat{\upbeta}}_E^{AB}=\frac{\sum_{ij}{Y}_{ij}{Z}_{ij}}{\sum_{ij}{Z}_{ij}}-\frac{\sum_{ij}{Y}_{ij}\left(1-{Z}_{ij}\right)}{\sum_{ij}\left(1-{Z}_{ij}\right)}$$

This estimator can be re-written to show that it compares intervention vs. control for episodes with a common treatment history; because this broadly matches the estimand (with the key difference being the estimand is based on a comparison of potential outcomes from the same patient, whereas the estimator is based on a comparison of randomised groups), we expect this estimator to be unbiased.

#### Per-patient added-benefit estimator

The per-patient estimator can be obtained by weighting each patient by the inverse of their number of episodes, i.e. ${W}_i=\frac{1}{M_i}$:

2 $${\hat{\upbeta}}_P^{AB}=\frac{\sum_{ij}{W}_i{Y}_{ij}{Z}_{ij}}{\sum_{ij}{W}_i{Z}_{ij}}-\frac{\sum_{ij}{W}_i{Y}_{ij}\left(1-{Z}_{ij}\right)}{\sum_{ij}{W}_i\left(1-{Z}_{ij}\right)}$$

#### Per-episode policy-benefit estimator

The policy-benefit estimators can be obtained using a two-step approach. In the first step, a causal model is specified for the effect of treatment history on the potential outcomes; then in the second step, estimates of the difference in potential outcomes obtained in the first step are combined into an overall estimate. We note that the exact models used to calculate this effect can vary (particularly depending on the number of episodes each patient experiences, e.g. more complex models would be required for trials in which some patients experience >2 episodes). Here, we described the estimator we used in the setting where patients experience a maximum of two episodes.

In the first step, we used the following model:

3 $${Y}_{ij}=\upalpha +\upbeta {Z}_{ij}+\upgamma {Z}_{i,j-1}+\updelta {Z}_{ij}{Z}_{i,j-1}+{\beta}_{ep}{X}_{e{p}_{ij}}+{\upvarepsilon}_{ij}$$

where *Z*_*i*, *j* − 1_ is the treatment allocation in the previous episode (and is set to 0 for *j* = 1), and ${X}_{e{p}_{ij}}$ is an indicator for episode 2 (i.e. ${X}_{e{p}_{ij}}=1$ for episode 2, and 0 otherwise). This model allows the effect of the intervention in episode 1 to carry forward into episode 2 (the term γ), and for the intervention to get more (or less) effective the 2nd time it is used (the term δ).

Then, in the second step, we use the estimates $\hat{\upbeta}$, $\hat{\upgamma}$, and $\hat{\updelta}$ to calculate an overall estimate of the policy-benefit effect. Here, we estimate the difference in potential outcomes as $\hat{\upbeta}$ for all first episodes, and as $\hat{\upbeta}+\hat{\upgamma}+\hat{\updelta}$ for all second episodes. The formula for the overall estimate is:

$${\hat{\upbeta}}_E^{PB}=\frac{N_1}{M_T}\left(\hat{\upbeta}\right)+\frac{N_2}{M_T}\left(\hat{\upgamma}+\hat{\upbeta}+\hat{\updelta}\right)$$

This formula weights $\hat{\upbeta}$ and $\hat{\upbeta}+\hat{\upgamma}+\hat{\updelta}$ by the proportion of episodes to which they correspond. We have included the term ${X}_{e{p}_{ij}}$ in model [3] even though it is not used directly to estimate the treatment effect, as it is associated with *Z*_*i*, *j* − 1_, and so can act as a confounder if omitted from the model.

#### Per-patient policy-benefit estimator

The per-patient policy-benefit estimator is obtained in the same way as the per-episode policy-benefit estimator, except estimates from model [3] are obtained using weighted least-squares, where each patient is weighted by the inverse of their number of episodes, ${W}_i=\frac{1}{M_i}$. After obtaining estimates and calculating the difference in potential outcomes for each episode in the same manner as above, the overall treatment effect is calculated as:

$${\hat{\upbeta}}_P^{PB}=\frac{M_{T(1)}}{N_T}\left(\hat{\upbeta}\right)+\frac{M_{T(2)}}{N_T}\left(\frac{1}{2}\hat{\upbeta}+\left(\frac{1}{2}\right)\left(\hat{\upbeta}+\hat{\upgamma}+\hat{\updelta}\right)\right)$$

where *M*_*T*(*j*)_ represents the total number of patients for whom *M*_*i*_ = *j*. In this equation, $\frac{M_{T(1)}}{N_T}\left(\hat{\upbeta}\right)$ is the component for patients where *M*_*i*_ = 1, and $\frac{M_{T(2)}}{N_T}\left(\frac{1}{2}\hat{\upbeta}+\left(\frac{1}{2}\right)\left(\hat{\upbeta}+\hat{\upgamma}+\hat{\updelta}\right)\right)$ is the component for patients where *M*_*i*_ = 2 (with $\frac{1}{2}\hat{\upbeta}$ being the 1st episode component, and $\left(\frac{1}{2}\right)\left(\hat{\upbeta}+\hat{\upgamma}+\hat{\updelta}\right)$ being the 2nd episode component).

#### Performance measures

The main aim of this simulation study was to evaluate bias, though a secondary aim was to evaluate the coverage of 95% confidence intervals in settings where estimators were unbiased. We did not evaluate the precision of the different estimators, as each estimator addressed a different question and so precision is less relevant in deciding between them.

We measured bias as $E\left(\hat{\upbeta}\right)-\upbeta$, where $E\left(\hat{\upbeta}\right)$ represents the mean of the estimates across all simulation replications, and β represents the true value of the estimand. We compared each estimator against its corresponding estimand (i.e. ${\hat{\upbeta}}_E^{AB}$ vs. ${\upbeta}_E^{AB}$, ${\hat{\upbeta}}_P^{AB}$ vs. ${\upbeta}_P^{AB}$, etc). We considered any estimator where the Monte Carlo standard error 95% confidence interval for bias (described below) did not include 0 (denoting unbiasedness) to be problematic.

We also evaluated coverage of the 95% confidence intervals. We defined coverage as the proportion of replications for which the 95% confidence interval of the estimator contained the true value of the estimand.

For each performance measure (bias, coverage) we also assessed the Monte Carlo standard error (MCSE), which provides a measure of variability for the estimated performance measure in the simulation study. We present the MCSEs as 95% confidence intervals alongside the mean bias and coverage, except in cases where this interval was too small to show up on the figure (i.e. when the width of the confidence interval was smaller than the size of the dot representing the mean bias or coverage), in which case we report the range of Monte Carlo standard errors for each performance measure across scenarios.

We used 10,000 replications for all simulation scenarios.

### Simulation study 1: patients enrolled for all episodes they experience

#### Data generating methods

Simulation study 1 is based on a trial of 300 patients; 150 patients experience one episode during the trial period, and 150 experience two episodes (i.e. *N*_*T*_ = 300, *M*_*T*_ = 450, *M*_*T*(1)_ = 150, and *M*_*T*(2)_ = 150.

The main purpose of this simulation study is to evaluate estimators in the setting where patients are enrolled for all episodes they experience; that is, the 150 patients who experienced two episodes were enrolled in the trial for both episodes (i.e. there are no patients who do not re-enrol for their 2nd episode).

We consider six different data generating mechanisms (described further below); all were based on the following general model for a continuous outcome:

5 $${Y}_{ij}=\upalpha +{\upbeta}_{trt}{Z}_{ij}+{\upbeta}_{ep}{X}_{e{p}_{ij}}+{\upbeta}_M{X}_{M_i}+{\upbeta}_{TRTxEP}{Z}_{ij}{X}_{e{p}_{ij}}+{\upbeta}_{TRTxM}{Z}_{ij}{X}_{M_i}+\upgamma {Z}_{i,j-1}+\updelta {Z}_{ij}{Z}_{i,j-1}+{\upmu}_i+{\upvarepsilon}_{ij}$$

where ${X}_{e{p}_{ij}}$ is an indicator variable for episode 2, and ${X}_{M_i}$ is an indicator variable for patients with *M*_*i*_ = 2. A description of the variables in this model are given in Table 3 (this table also contains some variables which are not in eq. (5), but are used in simulation studies 2a and 2b. Higher values of the outcome are better.

**Table 3:** Description of variables used in simulation study 1

Variable	Description	Method of generation
*Y*_*ij*_	Continuous outcome for patient *i* in episode *j*	Generated based on model [5]
*Z*_*ij*_	Treatment allocation (0 = control, 1 = intervention) for patient *i* in episode *j*	Bernoulli random variable with probability of 0.5 (implying simple randomisation
${X}_{e{p}_{ij}}$	Indicator for episode 2	NA
${X}_{M_i}$	Indicator for number of episodes patient experiences (0 = 1 episode, 1 = 2 episodes); equivalent to *M*_*i*_	NA
*Z*_*i*, *j* − 1_	Treatment allocation for patient *i* in episode *j* − 1; equal to 0 for episode 1	NA
${X}_{P{L}_i}$	Unobserved patient-level binary covariate, which is constant across episodes	Bernoulli random variable with probability of 0.5
${X}_{E{L}_{ij}}$	Unobserved episode-level binary covariate, which can vary across episodes	Bernoulli random variable with probability of 0.5
μ_*i*_	Random intercept for patient *i*	$\sim N\left(0,{\upsigma}_{\upmu}^2\right)$
ε_*ij*_	Random error term for episode *j* in patient *i*	$\sim N\left(0,{\upsigma}_{\upvarepsilon}^2\right)$

The parameter α is an intercept, β_*ep*_ and β_*M*_ are the effects of episode 2 and patient type (whether they experience 1 vs. 2 episodes) on outcome, and β_*trt*_, β_*TRTxEP*_, β_*TRTxM*_, γ, and δ are components of the treatment effect (e.g. β_*TRTxEP*_ is the interaction between treatment allocation and episode number, β_*TRTxM*_ is the interaction between treatment and patient type, and δ is the interaction between treatment allocation in the current episode and allocation in the previous episode).

In this study, we considered six different treatment effect mechanisms. This involved varying the parameters that define the treatment effect (β_*trt*_, β_*TRTxEP*_, β_*TRTxM*_, γ, and δ); values of these parameters for each scenario are shown in Table 4, along with the values of the four estimands for each scenario. For each scenario, we set α = 0, β_*trt*_ = 3, β_*ep*_ = 1, β_*M*_ = 1, ${\upsigma}_{\upmu}^2=5$ and ${\upsigma}_{\upvarepsilon}^2=5$. We generated μ_*i*_ and ε_*ij*_ independently; based on the chosen variances, the intraclass correlation between episodes from the same patient is 0.50 (conditional on the other variables in the data generating model).

**Table 4:** Simulation parameters and estimands for different scenarios in simulation study 1. For all scenarios, we set α = 0, β_*trt*_ = 3, β_*ep*_ = 1, β_*M*_ = 1, ${\upsigma}_{\upmu}^2=5$ and ${\upsigma}_{\upvarepsilon}^2=5$

	Parameters				Estimand values			
Scenario	β_*TRTxEP*_	β_*TRTxM*_	γ	δ	${\upbeta}_E^{AB}$	${\upbeta}_P^{AB}$	${\upbeta}_E^{PB}$	${\upbeta}_P^{PB}$
Scenario 1: Constant treatment effect	0	0	0	0	3	3	3	3
Scenario 2: Treatment effect varies across episode	1.5	0	0	0	3.5	3.38	3.5	3.38
Scenario 3: Treatment effect varies across patients with different values of M_*i*_	0	3	0	0	5	4.5	5	4.5
Scenario 4: Treatment effect carries forward	0	0	1	0	3	3	3.33	3.25
Scenario 5: Treatment becomes less effective on re-use	0	0	0	-3	2.5	2.63	2	2.25
Scenario 6: Treatment effect varies across episodes, across patients with different values of M_*i*_, carries forward, and becomes less effective on re-use	1,5	3	1	-3	5	4.5	4.83	4.38

We considered the following treatment effect mechanisms:

1. Constant treatment effect: the treatment effect is the same (β_*trt*_) across all episodes and patients
2. Treatment effect varies across episode: the treatment effect is different in the 1st episode (β_*trt*_) vs. in the 2nd episode (β_*trt*_ + β_*TRTxEP*_)
3. Treatment effect varies across patients with different values of *M*_*i*_: the treatment effect is different in patients who experience 1 episode (β_*trt*_) vs. those who experience 2 episodes (β_*trt*_ + β_*TRTxM*_).
4. Treatment effect carries forward into the 2nd episode: patients who receive intervention in the first episode have better outcomes in their 2nd episode (by the amount γ)
5. Treatment becomes less effective on re-use: patients receiving the intervention for the 1st time have a different treatment effect (β_*trt*_) than those receiving the intervention for the 2nd time (β_*trt*_ + δ)
6. Treatment effect varies across episodes, across patients with different values of *M*_*i*_, carries forward, and becomes less effective on re-use: the treatment effect is β_*trt*_ for patients who experience one episode. For patients who experience two episodes, the treatment effect is β_*trt*_ + β_*TRTxM*_ in the 1st episode, β_*trt*_ + β_*TRTxM*_ + β_*TRTxEP*_ in the 2nd episode for patients receiving the intervention for the first time (i.e. who received control in their 1st episode), and β_*trt*_ + β_*TRTxM*_ + β_*TRTxEP*_ + δ in the 2nd episode for patients receiving the intervention for the 2nd time (i.e. received intervention in their 1st episode). Patients who receive the intervention in the first episode also have better outcomes in their 2nd episode, by the amount γ.

We note that under certain treatment effect mechanisms, the values of certain estimands will coincide. Briefly, the added-benefit and policy-benefit estimands will coincide when treatment history does not influence either the outcome or treatment effect in the current episode, and the per-episode and per-patient estimands will coincide when the cluster size is not informative [14–19], i.e. when a patient’s average treatment effect across episodes does not depend on the number of episodes they experience. For further details on when estimand values will coincide, see reference (1).

### Simulation study 2a: some patients do not re-enrol for their 2nd episode

#### Data generating methods

The main purpose of this simulation study is to evaluate estimators when some of the patients who experience two episodes do not re-enrol in the trial for their second episode. For example, this may occur if patients find the trial procedures, such as number of follow-up visits, too burdensome; if they were disappointed at their treatment allocation in the first episode; or they experienced a poor outcome in their first episode.

As before, this simulation study is based on a trial of 300 patients; 150 patients experience one episode during the trial period, and 150 experience two episodes. All patients enrol for their first episode, but a subset of patients who experience two episodes do not re-enrol for their second episode. Therefore, *N*_*T*_ = 300 and *M*_*T*(1)_ = 150, however *M*_*T*(2)_ < 150 and *M*_*T*_ < 450; the exact values of *M*_*T*(2)_ and *M*_*T*_ vary across simulation replications.

We simulated data by first generating outcomes for all 450 episodes (regardless of whether they were enrolled in the trial for their 2nd episode) using model [6] below, and then generated an indicator for each episode to denote whether it was enrolled in the trial or not using model [7] below. We then performed analysis only on the subset of enrolled episodes. We used six different treatment effect mechanisms (based on model [6] below) and five different non-enrolment mechanisms (based on model [7] below), leading to 6 × 5 = 30 total scenarios. The different treatment effect and non-enrolment scenarios are described below.

We generated continuous outcomes from the model:

6 $${Y}_{ij}=\upalpha +{\upbeta}_{trt}{Z}_{ij}+{\upbeta}_{ep}{X}_{e{p}_{ij}}+{\upbeta}_M{X}_{M_i}+{\upbeta}_{TRTxEP}{Z}_{ij}{X}_{e{p}_{ij}}+{\upbeta}_{TRTxM}{Z}_{ij}{X}_{M_i}+\upgamma {Z}_{i,j-1}+\updelta {Z}_{ij}{Z}_{i,j-1}+{\upbeta}_{X_{PL}}{X}_{P{L}_i}+{\upbeta}_{X_{EL}}{X}_{E{L}_{ij}}+{\upmu}_i+{\upvarepsilon}_{ij}$$

This model is identical to model [5] from simulation study 1, except it contains two additional terms: ${X}_{P{L}_i}$ and ${X}_{E{L}_{ij}}$, which are unobserved binary covariates, with ${X}_{P{L}_i}$ being a patient-level covariate which does not vary across episodes, and ${X}_{E{L}_{ij}}$ being an episode-level covariate which can vary across episodes for the same patient; we use the subscript *PL* to denote ‘patient-level’, and *EL* to denote ‘episode-level’. The purpose of including ${X}_{P{L}_i}$ and ${X}_{E{L}_{ij}}$ in this model is to allow differential non-enrolment to be generated (this is explained further below). We used positive values for ${\upbeta}_{X_{PL}}$ and ${\upbeta}_{X_{EL}}$, so that patients or episodes where ${X}_{P{L}_i}=1$ or ${X}_{E{L}_{ij}}=1$ have better outcomes than if ${X}_{P{L}_i}$ or ${X}_{E{L}_{ij}}$ are 0; exact values of ${\upbeta}_{X_{PL}}$ and ${\upbeta}_{X_{EL}}$ for each scenario are shown in Table 5.

**Table 5:** Parameters for different episode 2 non-enrolment scenarios (simulation study 2a). For all scenarios, we set ${\upalpha}^{R_2}=0.05$ and ${\upgamma}^{R_2}=0.10$

Scenario	${\upbeta}_{X_{PL}}$	${\upbeta}_{X_{EL}}$	${\upbeta}_{X_{PL}}^{R_2}$	${\upbeta}_{X_{EL}}^{R_2}$	${\updelta}_{Xpl}^{R_2}$	${\updelta}_{Xel}^{R_2}$
Scenario 1 – Non-enrolment depends on previous treatment allocation	0	0	0	0	0	0
Scenario 2 – Non-enrolment depends on previous treatment allocation and previous outcome	10	0	0.25	0	0	0
Scenario 3 – Non-enrolment depends on previous treatment allocation and baseline prognosis at episode 2	0	10	0	0.25	0	0
Scenario 4 – Non-enrolment is differential between treatment groups based on previous outcome	10	0	0	0	0.5	0
Scenario 5 – Non-enrolment is differential between treatment groups based on baseline prognosis at episode 2	0	10	0	0	0	0.5

In the subset of patients with two episodes, we generated each patient’s probability of not re-enrolling for the second episode on a linear scale using the following model:

7 $$P\left({R}_{i2}=0\right)={\upalpha}^{R_2}+{\upgamma}^{R_2}{Z}_{i,j-1}+{\upbeta}_{X_{PL}}^{R_2}{X}_{P{L}_i}+{\upbeta}_{X_{EL}}^{R_2}{X}_{E{L}_{i2}}+{\updelta}_{Xpl}^{R_2}{Z}_{i,j-1}{X}_{P{L}_i}+{\updelta}_{Xel}^{R_2}{Z}_{i,j-1}{X}_{E{L}_{i2}}$$

where *R*_*ij*_ denotes whether patient *i* was enrolled for their *j* th episode (0 = not enrolled, 1 = enrolled). Note that *R*_*i*2_ = 0 for patients who only experience one episode, and *R*_*i*1_ = 1 for all patients. We use the superscript *R*_2_ for parameters to indicate that these parameters relate to the probability of not being re-enrolled for the 2nd episode. We set ${\upalpha}^{R_2}=0.05$ and ${\upgamma}^{R_2}=0.10$ for all scenarios. This implies that all patients have a non-zero probability of not re-enrolling for their second episode, and that patients who received intervention in episode 1 are more likely to not re-enrol than those in the control group (irrespective of ${X}_{P{L}_i}$ and ${X}_{E{L}_{i2}}$). Values for other parameters are shown in Table 5.

In the models above, we use ${X}_{P{L}_i}$ as a marker of the patient’s outcome in episode 1, and ${X}_{E{L}_{i2}}$ as a marker for the patient’s expected outcome in episode 2; that is, larger values of ${\upbeta}_{X_{PL}}^{R_2}$ denote that patients with better outcomes in episode 1 are less likely to re-enrol in the trial for their 2nd episode, and larger values of ${\upbeta}_{X_{EL}}^{R_2}$ denote that patients with better expected outcomes in episode 2 are less likely to re-enrol for that episode.

As stated above, we used six treatment effect mechanisms and five non-enrolment mechanisms. We used the same six treatment effect mechanisms as used in simulation study 1 (shown in Table 4), apart from the addition of ${X}_{P{L}_i}$ and ${X}_{E{L}_{ij}}$ to the model (as shown in model [6]). All other parameter values were the same as in Table 4, though the estimand values differed (these are described below). The five non-enrolment scenarios are shown in Table 5.

For each scenario, we calculated estimands based on the set of episodes enrolled in the trial. We calculated the true value of each estimand by generating a single large dataset of 1,000,000 patients (1,500,000 episodes), and then excluding episodes according to model [7] above. We then generated both added-benefit and policy-benefit potential treatment effects for each episode, and calculated the true value of the relevant estimand based on these. These estimand values are shown in the supplementary material.

### Simulation study 2b: further exploring bias associated with per-patient and policy-benefit estimators under non-enrolment scenarios 4 and 5

We developed simulation study 2b to further explore some of the results from simulation study 2a, pertaining to bias in the per-patient and policy-benefit estimators under certain non-enrolment mechanisms. We generated outcomes and non-enrolment in the same way as in simulation study 2a, but used a wider range of parameter values in order to assess how large the relevant parameter values needed to be in order for bias to become apparent. Full details of the data generation methods, parameter values, and results are available in the supplementary material.

## Results

### Simulation study 1: patients enrolled for all episodes they experience

**Fig. 1:**
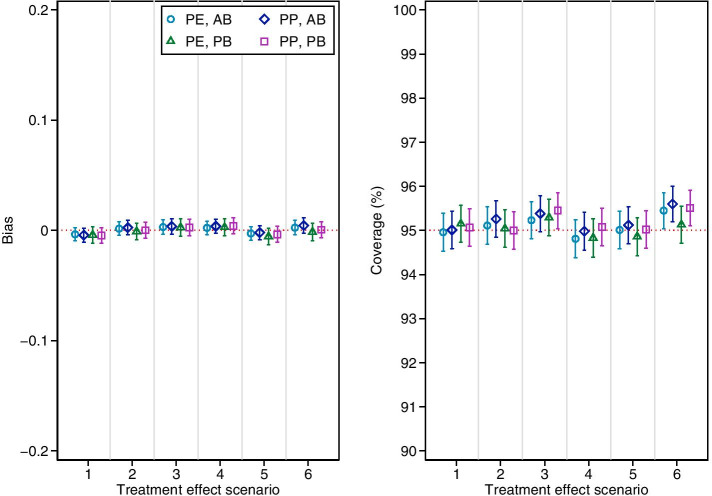
Bias and coverage of independence estimators in simulation study 1. PE = per-episode; PP = per-patient; AB = added-benefit; PB = policy-benefit. Error bars are 95% confidence intervals based on Monte Carlo standard errors. Scenario 1: Constant treatment effect. Scenario 2: Treatment effect varies across episode. Scenario 3: Treatment effect varies across patients with different values of *M*_*i*_. Scenario 4: Treatment effect carries forward. Scenario 5: Treatment becomes less effective on re-use. Scenario 6: Treatment effect varies across episodes, across patients with different values of *M*_*i*_, carries forward, and becomes less effective on re-use

Results are shown in Fig. 1. All estimators were unbiased and provided close to nominal coverage in all scenarios.

### Simulation study 2a: some patients do not re-enrol for their 2nd episode

**Fig. 2:**
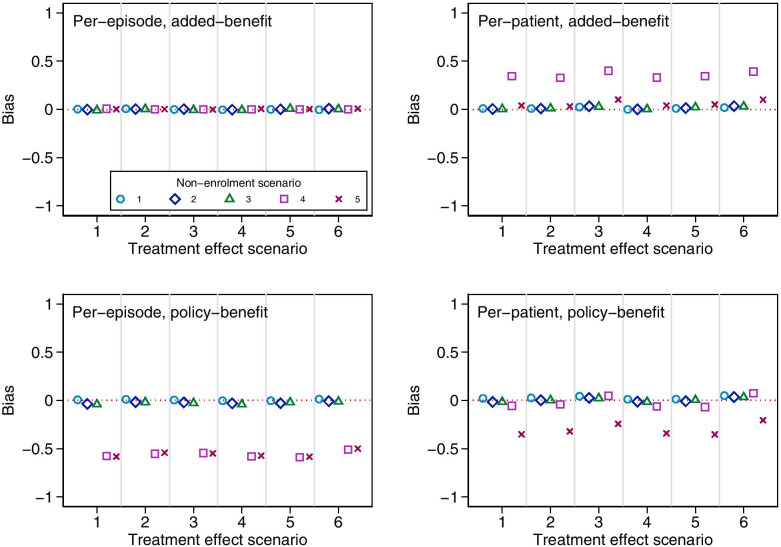
Bias in estimators across different treatment effect and non-enrolment scenarios for simulation study 2a. Monte Carlo standard errors ranges: per-episode added-benefit 0.003–0.006; per-episode policy-benefit 0.004–0.008; per-patient added-benefit 0.003–0.006; per-patient policy-benefit 0.004–0.007. Treatment effect scenario 1: Constant treatment effect. 2: Treatment effect varies across episode. 3: Treatment effect varies across patients with different values of *M*_*i*_. 4: Treatment effect carries forward. 5: Treatment becomes less effective on re-use. 6: Treatment effect varies across episodes, across patients with different values of *M*_*i*_, carries forward, and becomes less effective on re-use. Non-enrolment scenario 1: non-enrolment depends on previous treatment allocation. 2: Non-enrolment depends on previous treatment allocation and previous outcome. 3: Non-enrolment depends on previous treatment allocation and baseline prognosis at episode two. 4: Non-enrolment is differential between treatment groups based on previous outcome. 5: Non-enrolment is differential between treatment groups based on baseline prognosis at episode two

**Fig. 3:**
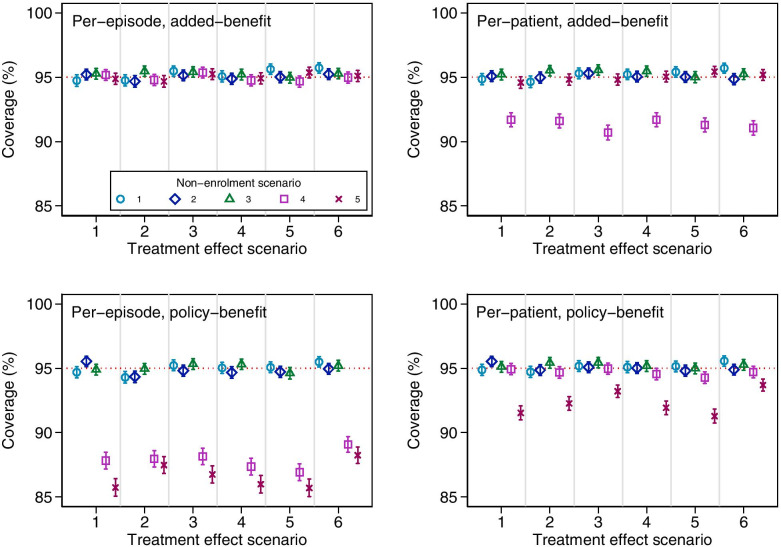
Coverage of estimators across different treatment effect and non-enrolment scenarios for simulation study 2a. Error bars are 95% confidence intervals based on Monte Carlo standard errors. Treatment effect scenario 1: Constant treatment effect. 2: Treatment effect varies across episode. 3: Treatment effect varies across patients with different values of *M*_*i*_. 4: Treatment effect carries forward. 5: Treatment becomes less effective on re-use. 6: Treatment effect varies across episodes, across patients with different values of *M*_*i*_, carries forward, and becomes less effective on re-use. Non-enrolment scenario 1: non-enrolment depends on previous treatment allocation. 2: Non-enrolment depends on previous treatment allocation and previous outcome. 3: Non-enrolment depends on previous treatment allocation and baseline prognosis at episode two. 4: Non-enrolment is differential between treatment groups based on previous outcome. 5: Non-enrolment is differential between treatment groups based on baseline prognosis at episode two

Results are shown in Figs. 2 and 3. The per-episode added-benefit estimator was unbiased across all scenarios, and had close to nominal coverage. The per-patient and policy-benefit estimators were unbiased across most scenarios, however, we identified several sources of bias which we discuss further below. Coverage of 95% confidence intervals was close to nominal for all settings in which estimators were unbiased.

The per-patient added-benefit estimator was biased for non-enrolment scenario 4, where non-enrolment was differential across treatment groups based on previous outcome. We also identified a small bias in non-enrolment scenario 5 (where non-enrolment is differential across treatment groups based on prognosis at episode 2) under treatment effect scenarios 3 and 6 (when the size of the treatment effect varied across patients with different values of *M*_*i*_). This bias was much smaller than that seen in non-enrolment scenario 4, but may still be large enough to cause concern.

The policy-benefit estimators (both per-patient and per-episode) were biased in non-enrolment scenarios 4 and 5. This occurred despite the fact that these estimators correctly modelled the causal effect of the previous treatment allocation on the outcome and treatment effect. This bias was a result of the model providing biased estimates of the parameter γ in these scenarios (which represents the effect of the previous allocation on outcome); because this parameter is used to construct policy-benefit estimates, these in turn will also be biased.

In these scenarios, episode 1 intervention patients with good outcomes were less likely to re-enrol for episode 2. At episode 2 therefore, most patients with a good outcome would have been allocated control in the previous episode. This created a false association between previous treatment allocation and outcome, which led to biased estimates of *γ*.

Interestingly, the per-patient policy-benefit estimator had negligible bias for non-enrolment scenario 4; this is likely because the per-patient estimator is biased upwards in this scenario, and the policy-benefit estimator is biased downwards, and the two biases cancel each other to some degree; however, under different parameter values it is likely that one of the biases would overtake the other, and the estimator would be biased. This is explored further in simulation study 2b below.

### Simulation study 2b: further exploring bias associated with per-patient and policy-benefit estimators under non-enrolment scenarios 4 and 5

Full results are available in the supplementary material. Briefly, the policy-benefit estimators were biased when either ${X}_{P{L}_i}$ or ${X}_{E{L}_{ij}}$ had strong associations with both outcome and probability of non-enrolment. When either association was small, bias was minimal, except when the other association was extremely large.

Similarly, the per-patient added-benefit estimator was biased when ${X}_{P{L}_i}$ had a strong association with both outcome and probability of non-enrolment; when either of these associations were small, bias was negligible, except when the other association was extremely large.

Unlike in simulation study 2a, we found the per-patient policy-benefit estimator was biased in certain settings, indicating that the two competing biases will not always cancel out.

As expected, the per-episode added-benefit estimator was unbiased in all scenarios.

## Discussion

In this article we report results from a large simulation study evaluating the use of independence estimators in re-randomisation trials. We found that the per-episode added-benefit estimator was unbiased across all scenarios considered. The per-patient estimators and policy-benefit estimators were also unbiased under the assumption of no differential non-enrolment (provided the causal model was correctly specified for the policy-benefit estimator). Furthermore, we found that the use of a robust standard error provided close to nominal coverage in all settings where the estimator was unbiased. These results suggest that the re-randomisation design alongside an independence estimator is a potentially useful option to estimate relevant treatment effects in multi-episode settings, though if per-patient or policy-benefit estimators are used it may be useful to conduct sensitivity analyses to evaluate how robust results are to violations of the above assumptions [20]. Furthermore, since most analyses of re-randomisation trials have used an independence per-episode added-benefit estimator, this article provides reassurance that reported results from these trials are unbiased.

The results in this article are based on simulation, and so are limited to the specific simulation scenarios studied. We used a wide range of treatment effect mechanisms and non-enrolment scenarios, and the results agree with previous analytical results [1]. However, it is possible the results found here may not apply to other settings, for instance when the sample size is very small [4]. It would also be of interest to evaluate these methods in a re-analysis of a published re-randomisation trial. This design is still quite new, and there are few published trials in the literature. However, further methodological work showing the design can provide robust answers may lead to an increased uptake.

In this paper we focussed on the setting where the interventions under study do not affect whether future episodes occur. This is a plausible assumption for some trials (e.g. a trial of high-dose Ibuprofen to manage pain in acute sickle cell pain crises [21], where Ibuprofen will not influence whether participants experience subsequent pain episodes), but will almost certainly be false in other trials (e.g. a trial of in vitro fertilisation, where a treatment success precludes further treatment episodes). In other settings this assumption will be unknown (e.g. in a trial using a drug to treat symptoms from severe asthma exacerbations, where it is unknown whether the underlying mechanism of the intervention may delay or even prevent subsequent exacerbations). If treatment does affect the occurrence of subsequent episodes, then the policy-benefit and per-patient estimands defined in this paper are no longer valid and so estimation of these effects would lead to results with no clear interpretation. However, the per-episode added-benefit estimand still applies to the setting where treatment affects subsequent episodes [1]. Therefore, if re-randomisation is being used in a setting where it is possible that treatment may affect occurrence of future episodes, we recommend using a per-episode added-benefit treatment effect, to ensure results are valid and interpretable.

Further extensions to the work in this paper would be useful. In particular, it would be useful to compare re-randomisation to alternative designs. For instance, a cluster design, where participants are assigned to intervention for all episodes vs. control for all episodes, may be a more robust way to estimate policy-benefit estimands. However, cluster designs may be affected by selection bias, where patients decide not to re-enrol based on knowledge of which treatment group they are in. This is not an issue in re-randomisation trials, as they are randomised between treatments at each re-enrolment (though of course, participants may not re-enrol due to past treatments). Further work comparing these designs would therefore be useful.

It would also be useful to evaluate whether alternatives to independence estimators, such as mixed-effects models, are suitable. Previous research has found they can be biased when the treatment effect carries forward into subsequent episodes, however it is not known how well these models work under other treatment effect mechanisms, such as when the treatment effect varies across patients or across episodes. We only considered a setting where patients were enrolled for a maximum of two episodes; it would be useful to evaluate policy-benefit estimators in the setting where patients experience a larger number of episodes, particularly as specifying an appropriate causal model in these settings will be more challenging. We found that robust standard errors worked well in the settings considered, however our primary concern was bias of estimated, and we designed the simulation study with this in mind. Follow-up simulation studies designed to specifically address the issue of robust vs model-based standard errors would be useful. Finally, as discussed above, we only considered the setting where treatment allocation does not affect the occurrence of subsequent episodes; it would be of interest to extend the policy-benefit and per-patient estimands to settings where this is not the case.

## Conclusions

Careful choice of estimand can ensure re-randomisation trials are addressing clinically relevant questions. Independence estimators are a useful approach, and should be considered as the default estimator until the statistical properties of alternative estimators are thoroughly evaluated.

## Supplementary Information

**Additional file 1.**

**Additional file 2.**

## Data Availability

The Stata code used to generate and analyse data in the simulation study is available in supplementary material file 2.
