# Supplementary material for: Independence estimators for re-randomisation trials in multi-episode settings: a simulation study
Source: BMC Med Res Methodol. 2021 Oct 30;21:235. doi: 10.1186/s12874-021-01433-4 (PMC8557515; doi:10.1186/s12874-021-01433-4)

**Suppementary material for: *Independence estimators for re-randomisation trials in multi-episode settings: a simulation study***

Brennan C Kahan, Ian R White, Sandra Eldridge, Richard Hooper

**Estimand values for simulation study 2a**

Estimand values for simulation study 2a are shown in table S1.

**Table S1: Estimand values for simulation study 2a.** *Treatment effect mechanism*: S1 = Constant treatment effect, S2 = Treatment effect varies across episode, S3 = Treatment effect varies across patients with different values of $M_{i}$, S4 = Treatment effect carries forward, S5 = Treatment becomes less effective on re-use, S6 = Treatment effect varies across episodes, across patients with different values of $M_{i}$, carries forward, and becomes less effective on re-use. *Non-enrolment scenarios*: S1 = Non-enrolment depends on previous treatment allocation, S2 = Non-enrolment depends on previous treatment allocation and previous outcome, S3 = Non-enrolment depends on previous treatment allocation and baseline prognosis at episode 2, S4 = Non-enrolment is differential between treatment groups based on previous outcome, S5 = Non-enrolment is differential between treatment groups based on baseline prognosis at episode 2.

| Treatment effect mechanism | Non-enrolment scenario | $\beta_{E}^{AB}$ | $\beta_{P}^{AB}$ | $\beta_{E}^{PB}$ | $\beta_{P}^{PB}$ |
| --- | --- | --- | --- | --- | --- |
| S1 | S1 | 3 | 3 | 3 | 3 |
|  | S2 | 3 | 3 | 3 | 3 |
|  | S3 | 3 | 3 | 3 | 3 |
|  | S4 | 3 | 3 | 3 | 3 |
|  | S5 | 3 | 3 | 3 | 3 |
| S2 | S1 | 3.47 | 3.34 | 3.47 | 3.34 |
|  | S2 | 3.42 | 3.29 | 3.42 | 3.29 |
|  | S3 | 3.42 | 3.29 | 3.42 | 3.29 |
|  | S4 | 3.42 | 3.29 | 3.42 | 3.29 |
|  | S5 | 3.42 | 3.29 | 3.42 | 3.29 |
| S3 | S1 | 4.97 | 4.5 | 4.97 | 4.5 |
|  | S2 | 4.92 | 4.5 | 4.92 | 4.5 |
|  | S3 | 4.92 | 4.5 | 4.92 | 4.5 |
|  | S4 | 4.92 | 4.5 | 4.92 | 4.5 |
|  | S5 | 4.92 | 4.5 | 4.92 | 4.5 |
| S4 | S1 | 3 | 3 | 3.31 | 3.23 |
|  | S2 | 3 | 3 | 3.28 | 3.19 |
|  | S3 | 3 | 3 | 3.28 | 3.19 |
|  | S4 | 3 | 3 | 3.28 | 3.19 |
|  | S5 | 3 | 3 | 3.28 | 3.19 |
| S5 | S1 | 2.56 | 2.68 | 2.07 | 2.33 |
|  | S2 | 2.61 | 2.73 | 2.16 | 2.42 |
|  | S3 | 2.61 | 2.73 | 2.16 | 2.42 |
|  | S4 | 2.67 | 2.77 | 2.16 | 2.42 |
|  | S5 | 2.67 | 2.77 | 2.16 | 2.42 |
| S6 | S1 | 4.99 | 4.52 | 4.81 | 4.39 |
|  | S2 | 4.95 | 4.52 | 4.78 | 4.40 |
|  | S3 | 4.95 | 4.52 | 4.78 | 4.40 |
|  | S4 | 5.01 | 4.57 | 4.78 | 4.40 |
|  | S5 | 5.01 | 4.57 | 4.78 | 4.40 |

**Simulation study 2b: further exploring bias associated with per-patient and policy-benefit estimators under non-enrolment scenarios 4 and 5**

***Data generating methods***

In this simulation study, we further explored some of the bias from per-patient and policy-benefit estimators associated with non-enrolment. As in simulation study 2a, we generated outcomes according to the model:

$$Y_{ij}=\alpha+\beta_{trt}Z_{ij}+\beta_{ep}X_{ep_{ij}}+\beta_{M}X_{M_{i}}+\beta_{TRTxEP}Z_{ij}X_{ep_{ij}}+\beta_{TRTxM}Z_{ij}X_{M_{i}}+\gamma Z_{i,j-1}+\delta Z_{ij}Z_{i,j-1}+\beta_{X_{PL}}X_{PL_{i}}+\beta_{X_{EL}}X_{EL_{ij}}+\mu_{i}+\varepsilon_{ij}$$

and probability of non-enrolment according to the model:

$$P\left( R_{i2}=0 \right)=\alpha^{R_{2}}+\gamma^{R_{2}}Z_{i,j-1}+\beta_{X_{PL}}^{R_{2}}X_{PL_{i}}+\beta_{X_{EL}}^{R_{2}}X_{EL_{i2}}+\delta_{Xpl}^{R_{2}}Z_{i,j-1}X_{PL_{i}}+\delta_{Xel}^{R_{2}}Z_{i,j-1}X_{EL_{i2}}$$

In the previous simulation study (2a) we used fairly large values for parameters associated with non-enrolment. In this simulation study, we used a range of values in order to assess how large the relevant parameter values needed to be in order for bias to become apparent.

Because the treatment effect mechanism did not have a large impact on bias in most scenarios in simulation study 2a, we opted to use a single treatment effect mechanism here. We used the constant treatment effect model for all scenarios (treatment effect mechanism 1); as such, the value of all estimands in these scenarios was 3.

For non-enrolment scenario 4, we varied $\beta_{X_{PL}}$ and $\delta_{Xpl}^{R_{2}}$ (which represents the increase in the probability of non-enrolment for patients with $X_{PL}=1$) in a factorial manner; we varied $\beta_{X_{PL}}$ between 0, 2.5, 5, 7.5, and 10, and we varied $\delta_{Xpl}^{R_{2}}$ between 0, 0.1, 0.2, 0.3, 0.4, 0.5, 0.6, 0.7, and 0.8. This led to 5x9=45 scenarios. We set $\beta_{X_{EL}}=0$ for all scenarios.

For non-enrolment scenario 5, we varied $\beta_{X_{EL}}$ and $\delta_{Xel}^{R_{2}}$ in a factorial manner; we varied $\beta_{X_{EL}}$ between 0, 2.5, 5, 7.5, and 10, and we varied $\delta_{Xel}^{R_{2}}$ between 0, 0.1, 0.2, 0.3, 0.4, 0.5, 0.6, 0.7, and 0.8. This led to 5x9=45 scenarios. We set $\beta_{X_{PL}}=0$ for all scenarios.

***Results***

Results are shown in figures S1-S4. The policy-benefit estimators were biased when either $X_{PL_{i}}$ or $X_{EL_{ij}}$ had strong associations with both outcome and probability of non-enrolment. When either association was small, bias was minimal, except when the other association was extremely large.

Similarly, the per-patient added-benefit estimator was biased when $X_{PL_{i}}$ had a strong association with both outcome and probability of non-enrolment; when either of these associations were small, bias was negligible, except when the other association was extremely large.

Unlike in simulation study 2a, we found the per-patient policy-benefit estimator was biased in certain settings, indicating that the two competing biases will not always cancel out.

**Figure S1: Bias in different estimators across non-enrolment scenario 4 in simulation study 2b.** Monte Carlo standard errors ranges: per-episode added-benefit 0.003-0.006; per-episode policy-benefit 0.004-0.008; per-patient added-benefit 0.003-0.006; per-patient policy-benefit 0.004-0.007.


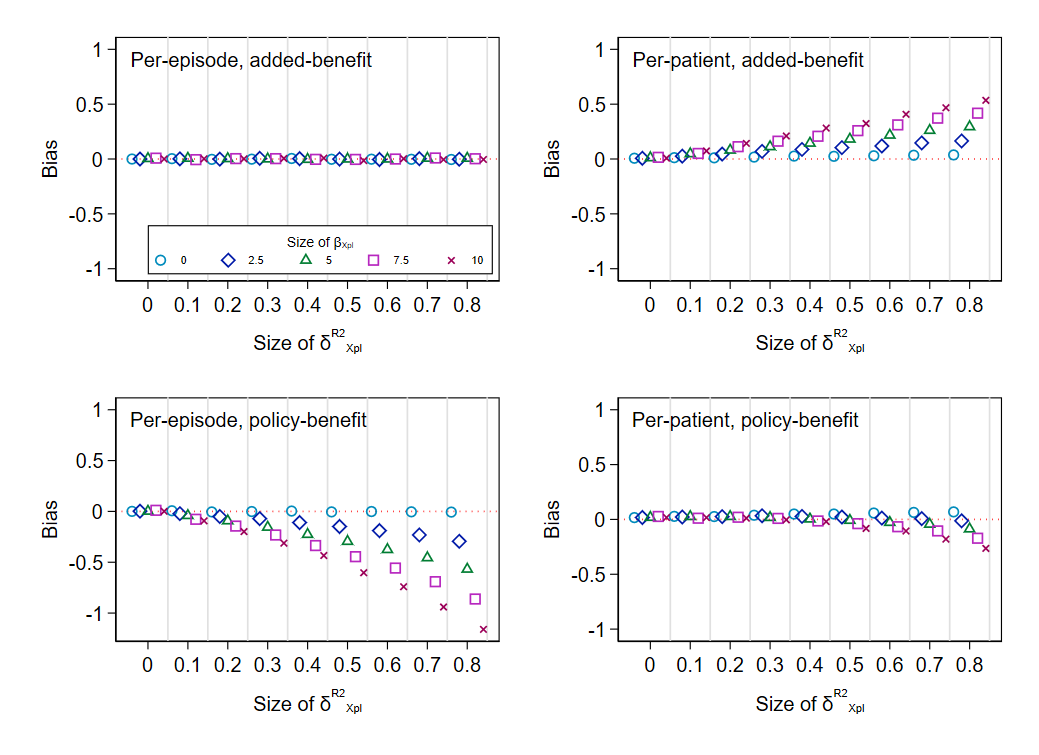


**Figure S2:** **Bias in different estimators across non-enrolment scenario 5 in simulation study 2b.** Monte Carlo standard errors ranges: per-episode added-benefit 0.003-0.006; per-episode policy-benefit 0.004-0.007; per-patient added-benefit 0.003-0.006; per-patient policy-benefit 0.004-0.007.


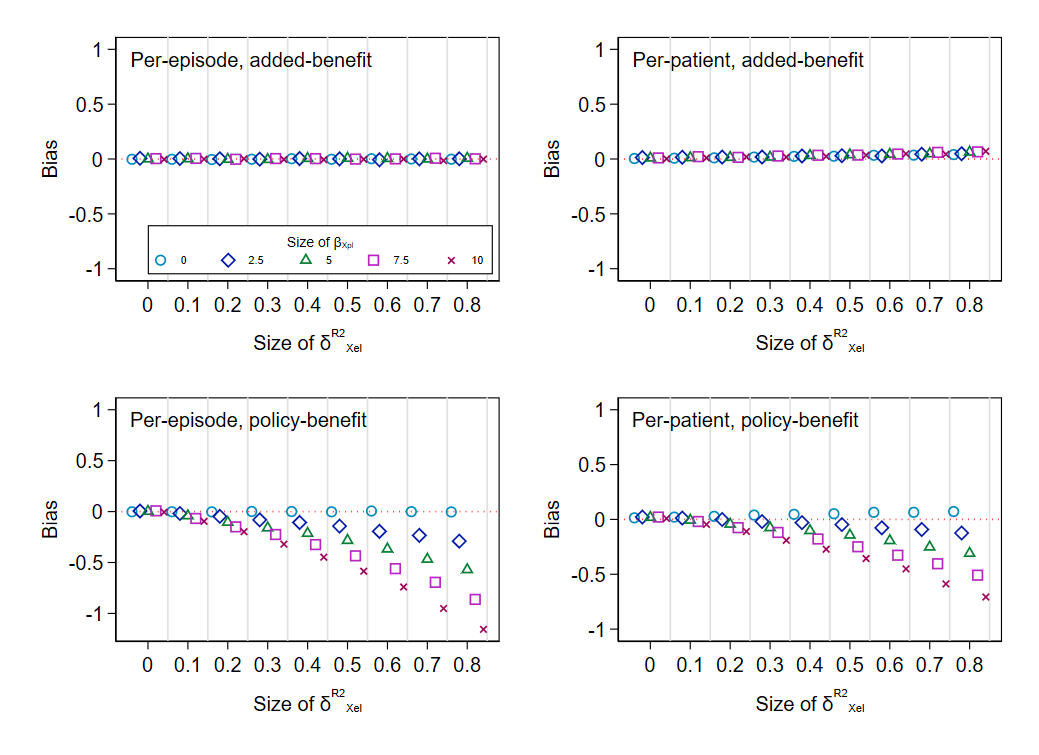


**Figure S3: Coverage of different estimators across non-enrolment scenario 4 in simulation study 2b.** Monte Carlo standard errors ranges: per-episode added-benefit 0.2-0.2; per-episode policy-benefit 0.2-0.5; per-patient added-benefit 0.2-0.3; per-patient policy-benefit 0.2-0.3.


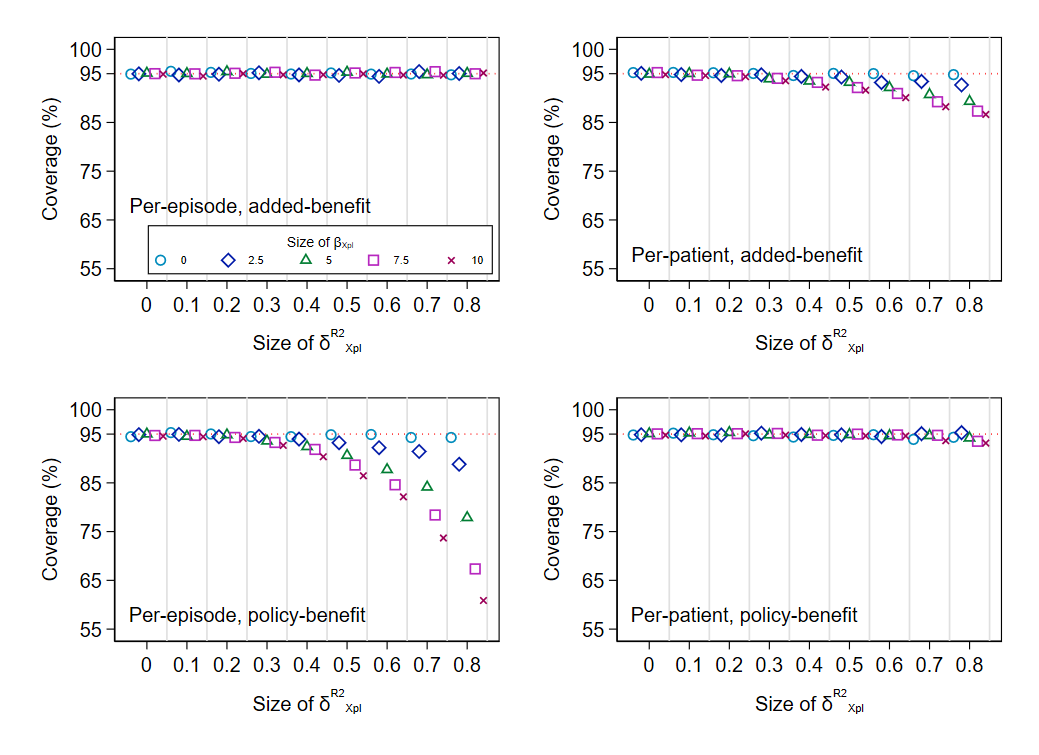


**Figure S4: Coverage of different estimators across non-enrolment scenario 5 in simulation study 2b.** Monte Carlo standard errors ranges: per-episode added-benefit 0.2-0.2; per-episode policy-benefit 0.2-0.5; per-patient added-benefit 0.2-0.2; per-patient policy-benefit 0.2-0.4.


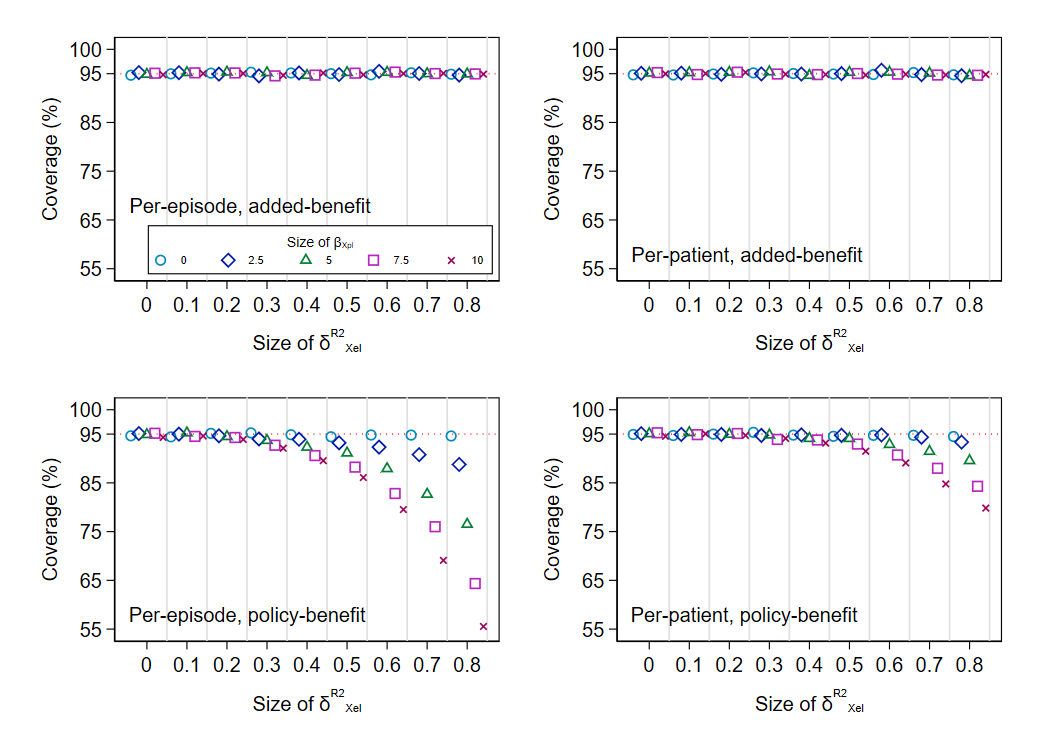

Supplement: Supplementary file 1 — Additional file 1. [file 12874_2021_1433_MOESM1_ESM.docx]
