# Supplementary material for: Independence estimators for re-randomisation trials in multi-episode settings: a simulation study
Source: BMC Med Res Methodol. 2021 Oct 30;21:235. doi: 10.1186/s12874-021-01433-4 (PMC8557515; doi:10.1186/s12874-021-01433-4)
Supplement: Supplementary file 2 — Additional file 2. [file 12874_2021_1433_MOESM2_ESM.docx]

**Suppementary material for: *Independence estimators for re-randomisation trials in multi-episode settings: a simulation study***

Brennan C Kahan, Ian R White, Sandra Eldridge, Richard Hooper

**Stata code used to generate data for simulation study 1**

clear all

*set trace on

set seed 19871

set more off

version 15.1

/**** setting up parameter values *****

*

**************************************/

*number of replications

local n_rep = 10000

*number of patients, proportion w/ 2 episodes

local n_patients = 300

local prop_2ep = 0.5

*variance parameters

local var_e = 5

local var_u = 5

*fixed regression parameters

local beta_trt = 3

local beta_ep = 1

local beta_pat = 1

/*** looping over trt eff scenarios 1-6 ***

*

******************************************/

foreach trt_eff_scenario in 1 2 3 4 5 6 {

if `trt_eff_scenario' == 1 {

local beta_prevtrt = 0

local beta_trtxep = 0

local beta_trtxpat = 0

local beta_trtxprevtrt = 0

}

else if `trt_eff_scenario' == 2 {

local beta_prevtrt = 0

local beta_trtxep = 1.5

local beta_trtxpat = 0

local beta_trtxprevtrt = 0

}

else if `trt_eff_scenario' == 3 {

local beta_prevtrt = 0

local beta_trtxep = 0

local beta_trtxpat = 3

local beta_trtxprevtrt = 0

}

else if `trt_eff_scenario' == 4 {

local beta_prevtrt = 1

local beta_trtxep = 0

local beta_trtxpat = 0

local beta_trtxprevtrt = 0

}

else if `trt_eff_scenario' == 5 {

local beta_prevtrt = 0

local beta_trtxep = 0

local beta_trtxpat = 0

local beta_trtxprevtrt = -3

}

else if `trt_eff_scenario' == 6 {

local beta_prevtrt = 1

local beta_trtxep = 1.5

local beta_trtxpat = 3

local beta_trtxprevtrt = -3

}

dis in red "trt_eff_scenario = `trt_eff_scenario'"

*** opening postfile ***

*

************************

cap postclose mysim

postfile mysim ///

///

trt_eff_scenario ///

beta_trt beta_prevtrt beta_trtxep beta_trtxpat beta_trtxprevtrt ///

///

n_patients prop_2ep ///

///

trt_eff_pe_ab ci_ll_pe_ab ci_ul_pe_ab ///

trt_eff_pp_ab ci_ll_pp_ab ci_ul_pp_ab ///

///

trt_eff_pe_pb ci_ll_pe_pb ci_ul_pe_pb ///

trt_eff_pp_pb ci_ll_pp_pb ci_ul_pp_pb ///

///

using "$replications\ch3 (ind est) - no non-enrolment - trt eff scenario `trt_eff_scenario'", replace

*for loop

forval i = 1/`n_rep' {

if mod(`i', 100) == 0 {

dis `i'

}

/**** setting up dataset ****

*

****************************/

clear

qui set obs `n_patients' // setting number of individual patients

qui gen id = _n // generating unique ID for patient

qui gen u = rnormal(0, sqrt(`var_u')) // generating random effect for patient

*generating number of patients w/ 1 vs 2 episodes

qui gen n_ep = 2 if id <= _N*`prop_2ep'

qui replace n_ep = 1 if n_ep != 2

/**** expanding to long-format *****/

qui expand n_ep

sort id

*generating episode number (1 or 2)

qui egen episode_number = seq(), from(1) by(id)

/*** generating variables required for data generating model ****/

*z (treatment allocation in current episode)

qui gen z = rbinomial(1, 0.5)

*z_prev (treatment allocation in previous episode)

qui gen z_prev = z[_n-1] if id[_n]==id[_n-1]

qui replace z_prev = 0 if episode_number == 1 // set to 0 if no previous episode

*x_pat

qui gen x_pat = 0 if n_ep == 1

qui replace x_pat = 1 if n_ep == 2

*x_ep

qui gen x_ep = episode_number - 1

*generating random effect for episode w/in patient

qui gen e = rnormal(0, sqrt(`var_e'))

/**** generating outcome ****

*

****************************/

qui gen y = `alpha' + `beta_trt'*z + `beta_ep'*x_ep + `beta_pat'*x_pat ///

+ `beta_prevtrt'*z_prev + `beta_trtxep'*z*x_ep + `beta_trtxpat'*z*x_pat ///

+ `beta_trtxprevtrt'*z*z_prev + u + e

/******************************* analysis *******************************

*

************************************************************************/

/****************** randomised-benefit *****************

*

*******************************************************/

/******** per-episode ********

*

*****************************/

qui reg y z, vce(cluster id)

matrix analysis_results = r(table)

local trt_eff_pe_ab = analysis_results[1,1] // treatment effect

local ci_ll_pe_ab = analysis_results[5,1] // lower limit 95% CI

local ci_ul_pe_ab = analysis_results[6,1] // upper limit 95% CI

/******** per-patient ********

*

*****************************/

qui reg y z [pw=1/n_ep], vce(cluster id)

matrix analysis_results = r(table)

local trt_eff_pp_ab = analysis_results[1,1] // treatment effect

local ci_ll_pp_ab = analysis_results[5,1] // lower limit 95% CI

local ci_ul_pp_ab = analysis_results[6,1] // upper limit 95% CI

/********************* policy-benefit *****************

*

*******************************************************/

/******** per-episode ********

*

*****************************/

*getting % of episodes which 1st ep, and % which are 2nd ep

qui summ x_ep

local prop_2nd_ep = r(mean)

local prop_1st_ep = 1 - r(mean)

qui reg y z##z_prev x_ep, vce(cluster id)

qui lincomest `prop_1st_ep'*_b[1.z] + `prop_2nd_ep'*(_b[1.z]+_b[1.z_prev] + _b[1.z#1.z_prev])

matrix analysis_results = r(table)

local trt_eff_pe_pb = analysis_results[1,1] // treatment effect

local ci_ll_pe_pb = analysis_results[5,1] // lower limit 95% CI

local ci_ul_pe_pb = analysis_results[6,1] // upper limit 95% CI

/******** per-patient ********

*

*****************************/

*getting % of patients w/ 1 episode vs 2 episodes

qui gen has_1ep = 1 if n_ep == 1

qui replace has_1ep = 0 if has_1ep != 1

qui replace has_1ep = . if episode_number == 2

qui summ has_1ep

local prop_has_1ep = r(mean)

local prop_has_2ep = 1 - r(mean)

qui reg y z##z_prev x_ep [pw=1/n_ep], vce(cluster id)

qui lincomest ///

`prop_has_1ep'*(_b[1.z]) + ///

`prop_has_2ep'*((1/2)*(_b[1.z]) + (1/2)*(_b[1.z]+_b[1.z_prev] + _b[1.z#1.z_prev]))

matrix analysis_results = r(table)

local trt_eff_pp_pb = analysis_results[1,1] // treatment effect

local ci_ll_pp_pb = analysis_results[5,1] // lower limit 95% CI

local ci_ul_pp_pb = analysis_results[6,1] // upper limit 95% CI

***** posting results to new dataset *****

*

******************************************

post mysim ///

///

(`trt_eff_scenario') ///

(`beta_trt') (`beta_prevtrt') (`beta_trtxep') (`beta_trtxpat') (`beta_trtxprevtrt') ///

///

(`n_patients') (`prop_2ep') ///

///

(`trt_eff_pe_ab') (`ci_ll_pe_ab') (`ci_ul_pe_ab') ///

(`trt_eff_pp_ab') (`ci_ll_pp_ab') (`ci_ul_pp_ab') ///

///

(`trt_eff_pe_pb') (`ci_ll_pe_pb') (`ci_ul_pe_pb') ///

(`trt_eff_pp_pb') (`ci_ll_pp_pb') (`ci_ul_pp_pb')

*closing for-loop

}

*closing postfile

postclose mysim

*closing foreach loop over trt effects scenarios

}

**Stata code used to generate data for simulation study 2a**

clear all

set seed 2030405

set more off

version 15.1

*set trace on

/**** setting up parameter values *****

*

**************************************/

*number of replications

local n_rep = 10000

*number of patients, proportion w/ 2 episodes

local n_patients = 300

local prop_2ep = 0.5

*variance parameters

local var_e = 5

local var_u = 5

*fixed regression parameters

local beta_trt = 3

local beta_ep = 1

local beta_pat = 1

*fixed regression parameters related to P(non-reenrolment) for 2nd episode

local pnr_alpha = 0.05 // baseline probability of non-return in 2nd episode

local pnr_beta_prev_z = 0.1 // effect of previous treatment on P(non-return)

*characteristics of full population of episodes

local m_total = `n_patients' + `n_patients'*`prop_2ep'

local n_t = `n_patients'

local m_t2 = `n_t'*`prop_2ep'

local m_t1 = `n_t'*(1-`prop_2ep')

/******* looping over treatment effect scenarios ****/

foreach trt_eff_scenario in 1 2 3 4 5 6 {

if `trt_eff_scenario' == 1 {

local beta_prevtrt = 0

local beta_trtxep = 0

local beta_trtxpat = 0

local beta_trtxprevtrt = 0

}

else if `trt_eff_scenario' == 2 {

local beta_prevtrt = 0

local beta_trtxep = 1.5

local beta_trtxpat = 0

local beta_trtxprevtrt = 0

}

else if `trt_eff_scenario' == 3 {

local beta_prevtrt = 0

local beta_trtxep = 0

local beta_trtxpat = 3

local beta_trtxprevtrt = 0

}

else if `trt_eff_scenario' == 4 {

local beta_prevtrt = 1

local beta_trtxep = 0

local beta_trtxpat = 0

local beta_trtxprevtrt = 0

}

else if `trt_eff_scenario' == 5 {

local beta_prevtrt = 0

local beta_trtxep = 0

local beta_trtxpat = 0

local beta_trtxprevtrt = -3

}

else if `trt_eff_scenario' == 6 {

local beta_prevtrt = 1

local beta_trtxep = 1.5

local beta_trtxpat = 3

local beta_trtxprevtrt = -3

}

/**** looping over P(non-re-enrolment) scenarios *****/

foreach p_non_reenrol_scenario in 1 2 3 4 5 {

if `p_non_reenrol_scenario' == 1 {

local pnr_beta_xconst = 0 // effect of x_xconst on P(non-return)

local pnr_beta_x_time_var = 0 // effect of x_time_var on P(non-return)

local pnr_beta_zprev_x_xconst = 0 // effect of z_prev##x_xconst interaction on P(non-return)

local pnr_beta_zprev_x_xtimevar= 0 // effect of z_prev##x_time_var interaction on P(non-return)

local beta_x_time_var_cov = 0

local beta_x_time_const_cov = 0

}

else if `p_non_reenrol_scenario' == 2 {

local pnr_beta_xconst = 0.25 // effect of x_xconst on P(non-return)

local pnr_beta_x_time_var = 0 // effect of x_time_var on P(non-return)

local pnr_beta_zprev_x_xconst = 0 // effect of z_prev##x_xconst interaction on P(non-return)

local pnr_beta_zprev_x_xtimevar= 0 // effect of z_prev##x_time_var interaction on P(non-return)

local beta_x_time_var_cov = 0

local beta_x_time_const_cov = 10

}

else if `p_non_reenrol_scenario' == 3 {

local pnr_beta_xconst = 0 // effect of x_xconst on P(non-return)

local pnr_beta_x_time_var = 0.25 // effect of x_time_var on P(non-return)

local pnr_beta_zprev_x_xconst = 0 // effect of z_prev##x_xconst interaction on P(non-return)

local pnr_beta_zprev_x_xtimevar= 0 // effect of z_prev##x_time_var interaction on P(non-return)

local beta_x_time_var_cov = 10

local beta_x_time_const_cov = 0

}

else if `p_non_reenrol_scenario' == 4 {

local pnr_beta_xconst = 0 // effect of x_xconst on P(non-return)

local pnr_beta_x_time_var = 0 // effect of x_time_var on P(non-return)

local pnr_beta_zprev_x_xconst = 0.5 // effect of z_prev##x_xconst interaction on P(non-return)

local pnr_beta_zprev_x_xtimevar= 0 // effect of z_prev##x_time_var interaction on P(non-return)

local beta_x_time_var_cov = 0

local beta_x_time_const_cov = 10

}

else if `p_non_reenrol_scenario' == 5 {

local pnr_beta_xconst = 0 // effect of x_xconst on P(non-return)

local pnr_beta_x_time_var = 0 // effect of x_time_var on P(non-return)

local pnr_beta_zprev_x_xconst = 0 // effect of z_prev##x_xconst interaction on P(non-return)

local pnr_beta_zprev_x_xtimevar= 0.5 // effect of z_prev##x_time_var interaction on P(non-return)

local beta_x_time_var_cov = 10

local beta_x_time_const_cov = 0

}

dis in red "trt_eff_scenario = `trt_eff_scenario' AND p_non_reenrol_scenario = `p_non_reenrol_scenario'"

*** opening postfile ***

*

************************

cap postclose mysim

postfile mysim ///

///

trt_eff_scenario p_non_reenrol_scenario ///

pnr_beta_xconst pnr_beta_x_time_var pnr_beta_zprev_x_xconst pnr_beta_zprev_x_xtimevar ///

///

n_t m_total m_t1 m_t2 ///

rr_n_t rr_m_total rr_m_t1 rr_m_t2 rr_num_2nd_ep ///

///

trt_eff_pe_ab ci_ll_pe_ab ci_ul_pe_ab ///

trt_eff_pp_ab ci_ll_pp_ab ci_ul_pp_ab ///

///

trt_eff_pe_pb ci_ll_pe_pb ci_ul_pe_pb ///

trt_eff_pp_pb ci_ll_pp_pb ci_ul_pp_pb ///

///

using "$replications\ch3 (ind est) - w non-enrolment - trt eff scenario - `trt_eff_scenario' - p_non_reenrol_scenario - `p_non_reenrol_scenario'", replace

*for loop

forval i = 1/`n_rep' {

if mod(`i', 100) == 0 {

dis `i'

}

qui {

/**** setting up dataset ****

*

****************************/

clear

qui set obs `n_patients' // setting number of individual patients

qui gen id = _n // generating unique ID for patient

qui gen u = rnormal(0, sqrt(`var_u')) // generating random effect for patient

qui gen x_constant_cov = rbinomial(1, 0.5) // constant (not time-verying) covariate for patients

*generating number of patients w/ 1 vs 2 episodes

qui gen n_ep = 2 if id <= _N*`prop_2ep'

qui replace n_ep = 1 if n_ep != 2

/**** expanding to long-format *****/

qui expand n_ep

sort id

*generating episode number (1 or 2)

qui egen episode_number = seq(), from(1) by(id)

/*** generating variables required for data generating model ****/

*z (treatment allocation in current episode)

qui gen z = rbinomial(1, 0.5)

*z_prev (treatment allocation in previous episode)

qui gen z_prev = z[_n-1] if id[_n]==id[_n-1]

qui replace z_prev = 0 if episode_number == 1 // set to 0 if no previous episode

*x_pat

qui gen x_pat = 0 if n_ep == 1

qui replace x_pat = 1 if n_ep == 2

*x_ep

qui gen x_ep = episode_number - 1

*x_time_var_cov

qui gen x_time_var_cov = rbinomial(1, 0.5)

*generating random effect for episode w/in patient

qui gen e = rnormal(0, sqrt(`var_e'))

/**** generating outcome ****

*

****************************/

qui gen y = `alpha' + `beta_trt'*z + `beta_ep'*x_ep + `beta_pat'*x_pat ///

+ `beta_x_time_var_cov'*x_time_var_cov + `beta_x_time_const_cov'*x_constant_cov ///

+ `beta_prevtrt'*z_prev + `beta_trtxep'*z*x_ep + `beta_trtxpat'*z*x_pat ///

+ `beta_trtxprevtrt'*z*z_prev + u + e

/***** generating P(non-return) *****

*

************************************/

qui gen prob_nonreturn = `pnr_alpha' + ///

`pnr_beta_prev_z'*z_prev + `pnr_beta_xconst'*x_constant_cov + `pnr_beta_x_time_var'*x_time_var_cov + ///

`pnr_beta_zprev_x_xconst'*z_prev*x_constant_cov + `pnr_beta_zprev_x_xtimevar'*z_prev*x_time_var_cov ///

if episode_number == 2

qui replace prob_nonreturn = 0.9999 if prob_nonreturn > 1 & episode_number == 2

qui gen nonreturn = rbinomial(1, prob_nonreturn) if episode_number == 2

*whether each episode was enrolled

gen enrolled = 1

replace enrolled = 0 if nonreturn == 1

*dropping episodes which were not enrolled

keep if enrolled == 1

/*** characteristics of trial-enrolled pop ****/

*number of enrolled episodes/patient

gen one = 1

egen rr_n_ep = total(one), by(id)

tab rr_n_ep if episode_number == 1

*total number of enrolled episodes

local rr_m_total = _N

*total number of enrolled patients

summ episode_number if episode_number == 1

local rr_n_t = r(N)

*number of enrolled patients who experience only one episode

summ episode_number if episode_number == 2 // number of episodes which are 2nd ep

local rr_num_2nd_ep = r(N)

local rr_m_t1 = `rr_n_t' - `rr_num_2nd_ep'

*number of enrolled patients who experience two episodes

local rr_m_t2 = `rr_num_2nd_ep'

/******************************* analysis *******************************

*

************************************************************************/

/****************** randomised-benefit *****************

*

*******************************************************/

/******** per-episode ********

*

*****************************/

qui reg y z, vce(cluster id)

matrix analysis_results = r(table)

local trt_eff_pe_ab = analysis_results[1,1] // treatment effect

local ci_ll_pe_ab = analysis_results[5,1] // lower limit 95% CI

local ci_ul_pe_ab = analysis_results[6,1] // upper limit 95% CI

/******** per-patient ********

*

*****************************/

qui reg y z [pw=1/rr_n_ep], vce(cluster id)

matrix analysis_results = r(table)

local trt_eff_pp_ab = analysis_results[1,1] // treatment effect

local ci_ll_pp_ab = analysis_results[5,1] // lower limit 95% CI

local ci_ul_pp_ab = analysis_results[6,1] // upper limit 95% CI

/********************* policy-benefit *****************

*

*******************************************************/

/******** per-episode ********

*

*****************************/

*getting % of episodes which 1st ep, and % which are 2nd ep

qui summ x_ep

local prop_2nd_ep = r(mean)

local prop_1st_ep = 1 - r(mean)

qui reg y z##z_prev x_ep, vce(cluster id)

qui lincomest `prop_1st_ep'*_b[1.z] + `prop_2nd_ep'*(_b[1.z]+_b[1.z_prev] + _b[1.z#1.z_prev])

matrix analysis_results = r(table)

local trt_eff_pe_pb = analysis_results[1,1] // treatment effect

local ci_ll_pe_pb = analysis_results[5,1] // lower limit 95% CI

local ci_ul_pe_pb = analysis_results[6,1] // upper limit 95% CI

/******** per-patient ********

*

*****************************/

*getting % of patients w/ 1 episode vs 2 episodes

qui gen has_1ep = 1 if rr_n_ep == 1

qui replace has_1ep = 0 if has_1ep != 1

qui replace has_1ep = . if episode_number == 2

qui summ has_1ep

local prop_has_1ep = r(mean)

local prop_has_2ep = 1 - r(mean)

qui reg y z##z_prev x_ep [pw=1/rr_n_ep], vce(cluster id)

qui lincomest ///

`prop_has_1ep'*(_b[1.z]) + ///

`prop_has_2ep'*((1/2)*(_b[1.z]) + (1/2)*(_b[1.z]+_b[1.z_prev] + _b[1.z#1.z_prev]))

matrix analysis_results = r(table)

local trt_eff_pp_pb = analysis_results[1,1] // treatment effect

local ci_ll_pp_pb = analysis_results[5,1] // lower limit 95% CI

local ci_ul_pp_pb = analysis_results[6,1] // upper limit 95% CI

*closing 'qui' option

}

***** posting results to new dataset *****

*

******************************************

post mysim ///

///

(`trt_eff_scenario') (`p_non_reenrol_scenario') ///

(`pnr_beta_xconst') (`pnr_beta_x_time_var') (`pnr_beta_zprev_x_xconst') (`pnr_beta_zprev_x_xtimevar') ///

///

(`n_t') (`m_total') (`m_t1') (`m_t2') ///

(`rr_n_t') (`rr_m_total') (`rr_m_t1') (`rr_m_t2') (`rr_num_2nd_ep') ///

///

(`trt_eff_pe_ab') (`ci_ll_pe_ab') (`ci_ul_pe_ab') ///

(`trt_eff_pp_ab') (`ci_ll_pp_ab') (`ci_ul_pp_ab') ///

///

(`trt_eff_pe_pb') (`ci_ll_pe_pb') (`ci_ul_pe_pb') ///

(`trt_eff_pp_pb') (`ci_ll_pp_pb') (`ci_ul_pp_pb')

*closing for-loop

}

*closing postfile

postclose mysim

*closing foreach loop

}

*closing 2nd foreach loop

}

**Stata code used to generate data for simulation study 2b**

clear all

set seed 3040506

set more off

version 15.1

*set trace on

/**** setting up parameter values *****

*

**************************************/

*number of replications

local n_rep = 10000

*number of patients, proportion w/ 2 episodes

local n_patients = 300

local prop_2ep = 0.5

*variance parameters

local var_e = 5

local var_u = 5

*fixed regression parameters

local beta_trt = 3

local beta_ep = 1

local beta_pat = 1

*regression parameters related to trt effect

local beta_prevtrt = 0

local beta_trtxep = 0

local beta_trtxpat = 0

local beta_trtxprevtrt = 0

*fixed regression parameters related to P(non-reenrolment) for 2nd episode

local pnr_alpha = 0.05 // baseline probability of non-return in 2nd episode

local pnr_beta_prev_z = 0.1 // effect of previous treatment on P(non-return)

*characteristics of full population of episodes

local m_total = `n_patients' + `n_patients'*`prop_2ep'

local n_t = `n_patients'

local m_t2 = `n_t'*`prop_2ep'

local m_t1 = `n_t'*(1-`prop_2ep')

/******* looping over effect of X on Y scenarios ****/

foreach x_vs_y_assocation in 0 2.5 5 7.5 10 {

/******* looping over P(non-return) scenarios ****/

foreach p_nonreturn in 0 10 20 30 40 50 60 70 80 {

/**** looping over P(non-re-enrolment) scenarios *****/

foreach p_non_reenrol_scenario in 4 5 {

if `p_non_reenrol_scenario' == 4 {

local pnr_beta_xconst = 0 // effect of x_xconst on P(non-return)

local pnr_beta_x_time_var = 0 // effect of x_time_var on P(non-return)

local pnr_beta_zprev_x_xconst = `p_nonreturn'/100 // effect of z_prev##x_xconst interaction on P(non-return)

local pnr_beta_zprev_x_xtimevar= 0 // effect of z_prev##x_time_var interaction on P(non-return)

local beta_x_time_var_cov = 0

local beta_x_time_const_cov = `x_vs_y_assocation'

}

else if `p_non_reenrol_scenario' == 5 {

local pnr_beta_xconst = 0 // effect of x_xconst on P(non-return)

local pnr_beta_x_time_var = 0 // effect of x_time_var on P(non-return)

local pnr_beta_zprev_x_xconst = 0 // effect of z_prev##x_xconst interaction on P(non-return)

local pnr_beta_zprev_x_xtimevar= `p_nonreturn'/100 // effect of z_prev##x_time_var interaction on P(non-return)

local beta_x_time_var_cov = `x_vs_y_assocation'

local beta_x_time_const_cov = 0

}

dis in red "x_vs_y_assocation = `x_vs_y_assocation'"

dis in red "p_nonreturn = `p_nonreturn'"

dis in red "p_non_reenrol_scenario = `p_non_reenrol_scenario'"

*** opening postfile ***

*

************************

cap postclose mysim

postfile mysim ///

///

p_non_reenrol_scenario p_nonreturn x_vs_y_assocation ///

pnr_beta_xconst pnr_beta_x_time_var pnr_beta_zprev_x_xconst pnr_beta_zprev_x_xtimevar ///

beta_x_time_var_cov beta_x_time_const_cov ///

///

n_t m_total m_t1 m_t2 ///

rr_n_t rr_m_total rr_m_t1 rr_m_t2 rr_num_2nd_ep ///

///

trt_eff_pe_ab ci_ll_pe_ab ci_ul_pe_ab ///

trt_eff_pp_ab ci_ll_pp_ab ci_ul_pp_ab ///

///

trt_eff_pe_pb ci_ll_pe_pb ci_ul_pe_pb ///

trt_eff_pp_pb ci_ll_pp_pb ci_ul_pp_pb ///

///

using "replications\ch3 (ind est) - exploring bias PP and PB - p_non_reenrol_scenario `p_non_reenrol_scenario' - p_nonreturn `p_nonreturn' - x_vs_y_assocation `x_vs_y_assocation'", replace

*for loop

forval i = 1/`n_rep' {

if mod(`i', 100) == 0 {

dis `i'

}

qui {

/**** setting up dataset ****

*

****************************/

clear

qui set obs `n_patients' // setting number of individual patients

qui gen id = _n // generating unique ID for patient

qui gen u = rnormal(0, sqrt(`var_u')) // generating random effect for patient

qui gen x_constant_cov = rbinomial(1, 0.5) // constant (not time-verying) covariate for patients

*generating number of patients w/ 1 vs 2 episodes

qui gen n_ep = 2 if id <= _N*`prop_2ep'

qui replace n_ep = 1 if n_ep != 2

/**** expanding to long-format *****/

qui expand n_ep

sort id

*generating episode number (1 or 2)

qui egen episode_number = seq(), from(1) by(id)

/*** generating variables required for data generating model ****/

*z (treatment allocation in current episode)

qui gen z = rbinomial(1, 0.5)

*z_prev (treatment allocation in previous episode)

qui gen z_prev = z[_n-1] if id[_n]==id[_n-1]

qui replace z_prev = 0 if episode_number == 1 // set to 0 if no previous episode

*x_pat

qui gen x_pat = 0 if n_ep == 1

qui replace x_pat = 1 if n_ep == 2

*x_ep

qui gen x_ep = episode_number - 1

*x_time_var_cov

qui gen x_time_var_cov = rbinomial(1, 0.5)

*generating random effect for episode w/in patient

qui gen e = rnormal(0, sqrt(`var_e'))

/**** generating outcome ****

*

****************************/

qui gen y = `alpha' + `beta_trt'*z + `beta_ep'*x_ep + `beta_pat'*x_pat ///

+ `beta_x_time_var_cov'*x_time_var_cov + `beta_x_time_const_cov'*x_constant_cov ///

+ `beta_prevtrt'*z_prev + `beta_trtxep'*z*x_ep + `beta_trtxpat'*z*x_pat ///

+ `beta_trtxprevtrt'*z*z_prev + u + e

/***** generating P(non-return) *****

*

************************************/

qui gen prob_nonreturn = `pnr_alpha' + ///

`pnr_beta_prev_z'*z_prev + `pnr_beta_xconst'*x_constant_cov + `pnr_beta_x_time_var'*x_time_var_cov + ///

`pnr_beta_zprev_x_xconst'*z_prev*x_constant_cov + `pnr_beta_zprev_x_xtimevar'*z_prev*x_time_var_cov ///

if episode_number == 2

qui replace prob_nonreturn = 0.0001 if prob_nonreturn < 0 & episode_number == 2

qui replace prob_nonreturn = 0.9999 if prob_nonreturn > 1 & episode_number == 2

qui gen nonreturn = rbinomial(1, prob_nonreturn) if episode_number == 2

*whether each episode was enrolled

gen enrolled = 1

replace enrolled = 0 if nonreturn == 1

*dropping episodes which were not enrolled

keep if enrolled == 1

/*** characteristics of trial-enrolled pop ****/

*number of enrolled episodes/patient

gen one = 1

egen rr_n_ep = total(one), by(id)

tab rr_n_ep if episode_number == 1

*total number of enrolled episodes

local rr_m_total = _N

*total number of enrolled patients

summ episode_number if episode_number == 1

local rr_n_t = r(N)

*number of enrolled patients who experience only one episode

summ episode_number if episode_number == 2 // number of episodes which are 2nd ep

local rr_num_2nd_ep = r(N)

local rr_m_t1 = `rr_n_t' - `rr_num_2nd_ep'

*number of enrolled patients who experience two episodes

local rr_m_t2 = `rr_num_2nd_ep'

/******************************* analysis *******************************

*

************************************************************************/

/****************** randomised-benefit *****************

*

*******************************************************/

/******** per-episode ********

*

*****************************/

qui reg y z, vce(cluster id)

matrix analysis_results = r(table)

local trt_eff_pe_ab = analysis_results[1,1] // treatment effect

local ci_ll_pe_ab = analysis_results[5,1] // lower limit 95% CI

local ci_ul_pe_ab = analysis_results[6,1] // upper limit 95% CI

/******** per-patient ********

*

*****************************/

qui reg y z [pw=1/rr_n_ep], vce(cluster id)

matrix analysis_results = r(table)

local trt_eff_pp_ab = analysis_results[1,1] // treatment effect

local ci_ll_pp_ab = analysis_results[5,1] // lower limit 95% CI

local ci_ul_pp_ab = analysis_results[6,1] // upper limit 95% CI

/********************* policy-benefit *****************

*

*******************************************************/

/******** per-episode ********

*

*****************************/

*getting % of episodes which 1st ep, and % which are 2nd ep

qui summ x_ep

local prop_2nd_ep = r(mean)

local prop_1st_ep = 1 - r(mean)

qui reg y z##z_prev x_ep, vce(cluster id)

qui lincomest `prop_1st_ep'*_b[1.z] + `prop_2nd_ep'*(_b[1.z]+_b[1.z_prev] + _b[1.z#1.z_prev])

matrix analysis_results = r(table)

local trt_eff_pe_pb = analysis_results[1,1] // treatment effect

local ci_ll_pe_pb = analysis_results[5,1] // lower limit 95% CI

local ci_ul_pe_pb = analysis_results[6,1] // upper limit 95% CI

/******** per-patient ********

*

*****************************/

*getting % of patients w/ 1 episode vs 2 episodes

qui gen has_1ep = 1 if rr_n_ep == 1

qui replace has_1ep = 0 if has_1ep != 1

qui replace has_1ep = . if episode_number == 2

qui summ has_1ep

local prop_has_1ep = r(mean)

local prop_has_2ep = 1 - r(mean)

qui reg y z##z_prev x_ep [pw=1/rr_n_ep], vce(cluster id)

qui lincomest ///

`prop_has_1ep'*(_b[1.z]) + ///

`prop_has_2ep'*((1/2)*(_b[1.z]) + (1/2)*(_b[1.z]+_b[1.z_prev] + _b[1.z#1.z_prev]))

matrix analysis_results = r(table)

local trt_eff_pp_pb = analysis_results[1,1] // treatment effect

local ci_ll_pp_pb = analysis_results[5,1] // lower limit 95% CI

local ci_ul_pp_pb = analysis_results[6,1] // upper limit 95% CI

*closing 'qui' option

}

***** posting results to new dataset *****

*

******************************************

post mysim ///

///

(`p_non_reenrol_scenario') (`p_nonreturn') (`x_vs_y_assocation') ///

(`pnr_beta_xconst') (`pnr_beta_x_time_var') (`pnr_beta_zprev_x_xconst') (`pnr_beta_zprev_x_xtimevar') ///

(`beta_x_time_var_cov') (`beta_x_time_const_cov') ///

(`n_t') (`m_total') (`m_t1') (`m_t2') ///

(`rr_n_t') (`rr_m_total') (`rr_m_t1') (`rr_m_t2') (`rr_num_2nd_ep') ///

///

(`trt_eff_pe_ab') (`ci_ll_pe_ab') (`ci_ul_pe_ab') ///

(`trt_eff_pp_ab') (`ci_ll_pp_ab') (`ci_ul_pp_ab') ///

///

(`trt_eff_pe_pb') (`ci_ll_pe_pb') (`ci_ul_pe_pb') ///

(`trt_eff_pp_pb') (`ci_ll_pp_pb') (`ci_ul_pp_pb')

*closing for-loop (replications)

}

*closing postfile

postclose mysim

*closing 1st loop

}

*closing 2nd loop

}

*closing 3rd loop

}
